# Underused capability and burnout: distributive-justice appraisal and psychological capital among Chinese public-sector employees

**DOI:** 10.3389/fpsyg.2026.1932134

**Published:** 2026-10-14

**Authors:** Zhe Cui, Chenxi Sun, Xinan Zhao

**Affiliations:** 1 School of Business Administration, Northeastern University, Shenyang, Liaoning, China; 2 School of Business, Shenyang University, Shenyang, Liaoning, China

**Keywords:** cynicism, emotional exhaustion, employee well-being, less favorable distributive-justice appraisal, perceived overqualification, psychological capital

## Abstract

**Background:**

Occupational burnout may co-occur with employees’ appraisal that their capability is underused and their contributions insufficiently recognized.

**Methods:**

Guided primarily by conservation of resources theory and social exchange theory, and situated within the broader Job Demands–Resources framework, this study analyzed a two-section survey of 415 employees from 21 Chinese public-sector organizations. Psychological capital (PsyCap) was assessed in the first section; perceived overqualification (POQ), less favorable distributive-justice appraisal (LF-DJA), and burnout were assessed together in the later section. A latent-variable structural equation model examined the core associations, with complementary interaction regressions and sensitivity analyses.

**Results:**

POQ showed positive total and LF-DJA-related indirect associations with emotional exhaustion and cynicism, but its residual direct associations were not statistically distinguishable from zero. Low professional efficacy (LPE) showed a different exploratory pattern. PsyCap most consistently conditioned the POQ–LF-DJA association. LF-DJA-related moderation depended on outcome and specification; conditional indirect associations were not supported in the model containing both outcome interaction terms. Procedure and occupational-context adjustment produced similar focal coefficients.

**Contribution:**

The findings distinguish capability-role appraisal, distributive-justice appraisal, and personal resources in an employee well-being account.

**Limitations:**

The contemporaneous assessment of POQ, LF-DJA, and burnout precludes temporal or causal conclusions; multiplicity and limited precision qualify weaker findings.

## 1 Introduction

Burnout is a central workplace well-being concern because it reflects employees’ exhaustion, detachment, and, for some employees, weakened efficacy self-evaluations (Demerouti et al., 2014; Maslach and Leiter, 2016). Although workload is a prominent correlate, burnout can also co-occur with employees’ appraisal that their capability is underused, their effort is insufficiently recognized, or their work provides limited meaningful contribution. The present study uses Chinese public-sector employment as a contextual backdrop for examining these employee-level appraisals. It does not treat public-sector governance as the focal explanatory construct; the empirical focus is how perceived overqualification (POQ), resource threat, reward-recognition appraisal, and well-being co-occur.

Qualification mismatch is a recurring concern in research on work and employment, and perceived overqualification is associated with employees’ job attitudes and experiences (Arvan et al., 2019; Harari et al., 2017; Maynard et al., 2006). The International Labour Office has documented skills and qualification mismatch, with overqualification representing one important form (International Labour Office, 2019). Reviews place overqualification within the broader literature on underemployment and person–job mismatch (McKee-Ryan and Harvey, 2011). Conceptually, POQ reflects a mismatch between employees’ knowledge, skills and abilities and the limited challenge provided by their jobs (Maynard et al., 2015; Maynard et al., 2006). It is therefore not merely individual dissatisfaction, but also a concern related to person–job fit, talent allocation, employee mental health and organizational performance (Erdogan and Bauer, 2021; Maynard et al., 2006).

China provides a useful setting because qualification mismatch is salient and the sample includes universities, state-owned enterprises, hospitals, and government agencies (Chen et al., 2017; Zheng et al., 2021). These work settings can combine high entry credentials with roles that employees may experience as repetitive, low in challenge, or limited in visible capability use. Such contextual features matter here only insofar as employees appraise their resources as underused, their prospects as constrained, or their work as insufficiently meaningful. The study therefore treats the public-sector setting as a backdrop for employee appraisal and workplace well-being, rather than as a test of public-administration arrangements.

From a conservation of resources perspective, employees may experience chronically underused knowledge, skills, and abilities as valued resources that are not being activated, replenished, or recognized (Cheng et al., 2021; Lin et al., 2022). This appraisal can be associated with lower motivation and meaning and with the burnout dimensions of emotional exhaustion and cynicism (Balch et al., 2011; Cao and Wei, 2022; Demerouti et al., 2014). Accordingly, this study considers whether capability underuse is associated with workplace well-being beyond the familiar workload account. Low professional efficacy (LPE) is examined separately because it is a competence-related self-evaluation that may not follow the same pattern.

Despite the prevalence of POQ, its consequences remain theoretically and empirically inconsistent, revealing a paradox in its effects (Erdogan and Bauer, 2021). One stream of research regards overqualification as a negative form of misfit, in which employees experience frustration, dissatisfaction, reduced motivation and weakened organizational identification because their abilities are not fully used (Cheng et al., 2020; Erdogan and Bauer, 2021; Maynard et al., 2006; Maynard et al., 2015; Maynard and Parfyonova, 2013). Another stream suggests that, when organizations provide sufficient resources, autonomy and developmental opportunities, employees’ surplus knowledge, skills and experience may be transformed into valuable organizational resources and may even promote positive outcomes (Deng et al., 2018; Luksyte and Spitzmueller, 2016; Zhang et al., 2021). These inconsistent findings suggest that overqualification may serve either as a burnout risk factor or as a potential resource, depending on the proposed explanatory processes and boundary conditions examined.

A less favorable distributive-justice appraisal (LF-DJA) is the focal reward-recognition appraisal in this study. It is operationalized by reverse-scoring four distributive-justice items, so higher scores indicate that employees appraise received outcomes as less commensurate with their contributions. This variable is not an independently measured circumstance of unfair outcome allocation, nor does it audit the fairness of any employer’s policies. It captures an employee-reported appraisal that may be relevant to resource threat, emotional exhaustion, cynicism, and workplace well-being (Cropanzano and Mitchell, 2005; Robbins et al., 2012).

POQ and LF-DJA can therefore be treated as related but distinct employee appraisals. POQ concerns perceived capability-role mismatch, whereas LF-DJA concerns the appraisal of outcome fairness relative to contribution. Employees who feel underused may also be more attentive to whether recognition, rewards, and opportunities appear commensurate with their inputs; conversely, a less favorable outcome appraisal may co-occur with stronger perceptions of resource threat. The present study does not assume a feedback sequence. It tests a theoretically ordered statistical association pattern while acknowledging that the appraisals may be mutually related or jointly shaped by unmeasured work conditions.

Prior research links qualification mismatch and outcome-fairness appraisals to strain-related work outcomes (Cheng et al., 2020; Creed and Blume, 2013; Erdogan and Bauer, 2021; Khan et al., 2024). Building on social exchange and conservation of resources reasoning, the present study asks whether POQ covaries with LF-DJA and whether LF-DJA statistically accounts for an indirect association between POQ and burnout. The formulation is intentionally associational because the core POQ-LF-DJA-burnout measures were assessed in the same later survey section.

Burnout deserves attention because it is closely associated with adverse mental and physical health outcomes (Thorsteinsson et al., 2014). The World Health Organization included burnout in ICD-11 as an occupational phenomenon under “factors influencing health status or contact with health services” (World Health Organization, 2019). The Job Demands–Resources model has become a dominant framework for explaining burnout because of its conceptual clarity and integrative capacity (Bakker and Demerouti, 2017), and has been widely applied to civil servants, teachers and healthcare workers (Cao et al., 2020; Gao, 2019; Mohammed et al., 2022; Schaufeli and Taris, 2014).

The Job Demands–Resources model provides a broader occupational-health context by distinguishing demanding work conditions and available resources (Bakker and Demerouti, 2017; Demerouti et al., 2001). The present study does not measure conventional job demands or job resources and therefore does not test the full JD-R process. COR is the primary basis for linking capability underuse and insufficient resource return with strain, whereas SET explains the relevance of employees’ input–outcome appraisal. This division of explanatory roles guides the model without treating the three frameworks as interchangeable.

To explain why employees do not respond uniformly to capability underuse or less favorable reward-recognition appraisals, the study includes psychological capital (PsyCap) as a conditional personal resource. PsyCap comprises hope, efficacy, resilience, and optimism (Luthans et al., 2007). Employees with stronger PsyCap may appraise resource threat differently and may show weaker associations between POQ, LF-DJA, and burnout outcomes (Dwyer et al., 2019; Luthans et al., 2005; Pang et al., 2021). This role is evaluated as a statistical condition, not as evidence that personal resources substitute for supportive work conditions.

The study addresses three linked questions. First, it examines whether POQ and LF-DJA are associated with emotional exhaustion and cynicism, the primary burnout outcomes. Overall burnout is reported as an auxiliary summary, and LPE is examined as an exploratory competence-related outcome. Second, it tests whether LF-DJA statistically accounts for indirect associations between POQ and these outcomes. Third, it examines whether PsyCap conditions the POQ-LF-DJA and burnout associations. The study provides employee-level association evidence and does not estimate organizational-level processes, objectively verify allocation decisions, or establish causal ordering.

This framing positions the study in organizational psychology. It distinguishes a capability-role appraisal (POQ), a reverse-scored outcome-fairness appraisal (LF-DJA), a personal-resource configuration (PsyCap), and employee well-being outcomes. The distinction matters because the same capability surplus may be experienced as a resource in a role offering discretion, development, and recognition but as a resource threat in a role where employees perceive capability underuse and weak return on contribution. By separating emotional exhaustion, cynicism, and LPE, the study also avoids treating burnout as a uniformly expressed condition.

The public-sector focus is analytically relevant because the sample spans several work settings in which employees may encounter a combination of professional credentials, stable roles, and varying opportunities to use and develop their capability. This contextual backdrop helps make POQ meaningful, but it is not modeled as a public-sector governance mechanism. The substantive analysis concerns employee appraisals and well-being within the sampled settings. Occupational context and organization membership are considered in sensitivity analyses, without treating these analyses as tests of institutional or sector-level causal effects.

A psychologically focused account is needed because POQ is an employee appraisal rather than an objective audit of credentials or job requirements. Employees in similar formal roles may differ in what they expect from challenge, recognition, development, and return on investment. Those differences can shape whether surplus capability is experienced as a usable resource or a resource threat. The present study therefore locates public-sector employment in the background and centers employee-level relationships among POQ, LF-DJA, PsyCap, emotional exhaustion, cynicism, overall burnout, and LPE.

## 2 Theoretical framework and hypotheses

### 2.1 Perceived overqualification, burnout, and resource-underutilization associations

Perceived overqualification (POQ) refers to employees’ subjective perception that their education, knowledge, skills, abilities, and experience exceed the requirements of their current job and are not fully utilized in their work (Erdogan et al., 2011; Maynard et al., 2015). In essence, POQ captures employees’ perception that their capability supply exceeds job demand (Liao et al., 2025; Maynard et al., 2006).

The concept of overqualification originates from the broader literature on underemployment. Early studies often linked overqualification to overeducation, emphasizing the mismatch between employees’ educational attainment and job requirements. As the concept evolved, scholars gradually distinguished between subjective and objective overqualification. Subjective overqualification focuses on employees’ psychological perception of the discrepancy between their qualifications and job requirements (Erdogan and Bauer, 2009, 2021; Maynard et al., 2006). Objective overqualification, in contrast, is assessed by comparing individuals’ actual qualifications with job requirements on the basis of external evaluations or objective indicators (Maltarich et al., 2011). However, because job requirements and work content are highly heterogeneous and difficult to quantify precisely, objective indicators may not fully capture employees’ lived psychological experiences in specific work contexts. Accordingly, recent studies have increasingly focused on subjective overqualification, namely POQ (Erdogan et al., 2011; Liao et al., 2025; Maynard et al., 2006).

This subjective approach is particularly useful for explaining employees’ psychological processes and work-related behaviors (Erdogan et al., 2011; Harari et al., 2017; Hu et al., 2015). By contrast, relying solely on objective qualification–job fit may overlook the subjective interpretations and complex psychological responses embedded in overqualification at the individual level (Maynard et al., 2015; Maynard and Parfyonova, 2013). Moreover, even employees holding the same formal position may differ substantially in their actual tasks, workload intensity, and role boundaries, which further limits the accuracy of objective measurement (Maltarich et al., 2011).

### 2.2 Perceived overqualification, burnout, and resource underutilization

In this study, we focus on an association pattern in which employees who perceive stronger overqualification also report less favorable outcome-fairness appraisals and burnout-related strain. When employees persistently perceive that their knowledge and abilities are not effectively used, and when their jobs fail to provide corresponding task challenge, developmental opportunities, or rewards, POQ is more likely to be experienced as resource waste and value underrecognition. Such perceptions may co-occur with less favorable fairness judgments regarding organizational rewards and higher burnout-related strain. Accordingly, this study treats POQ as a theoretically ordered employee appraisal in a statistical association model and examines whether LF-DJA statistically accounts for the indirect association between POQ and burnout outcomes. To avoid conceptual ambiguity, the term “overqualification” hereafter refers to perceived overqualification (POQ), rather than objective overqualification, unless otherwise specified.

Job burnout is generally defined as a persistent negative psychological syndrome that develops under prolonged work-related stress (Maslach et al., 2001). In contemporary research, burnout is regarded as a work-related syndrome resulting from chronic occupational stress rather than an immediate reaction to short-term stressful events. Maslach’s three-dimensional model has become one of the most widely used frameworks for conceptualizing burnout. In this model, burnout consists of emotional exhaustion, cynicism, and reduced professional efficacy (Maslach et al., 2001; Schaufeli et al., 1996). Emotional exhaustion refers to the depletion of employees’ emotional and energetic resources. Cynicism reflects a detached, indifferent, and negative attitude toward one’s work, work recipients, or organization. Reduced professional efficacy refers to employees’ negative evaluation of their competence, work ability, and work accomplishments.

The international relevance of burnout has been further highlighted by the World Health Organization. In the 11th Revision of the International Classification of Diseases (ICD-11), burnout is described as an occupational phenomenon resulting from chronic workplace stress that has not been successfully managed (World Health Organization, 2019). Its core characteristics include energy depletion or exhaustion, increased mental distance from one’s job or cynicism, and reduced professional efficacy. This international classification reinforces the relevance of Maslach’s three-dimensional framework and underscores burnout as an important occupational health concern.

Among the three dimensions, emotional exhaustion is generally considered the most central and immediate manifestation of burnout, whereas cynicism is often understood as a defensive psychological response that develops after prolonged resource depletion. By contrast, although reduced professional efficacy is also an important component of burnout, its relationship with antecedent variables may vary across research contexts (Maslach et al., 2001). This issue is particularly relevant in POQ research. Employees who perceive themselves as overqualified may experience ability underutilization and emotional exhaustion, while still maintaining a relatively high evaluation of their own competence (Ma et al., 2020; van Dijk et al., 2020; Wang, 2020; Zhang et al., 2016). Therefore, this study examines burnout both as an overall construct and at the dimensional level. In the empirical analysis, the professional efficacy dimension is reverse-coded and labeled as low professional efficacy (LPE), so that higher scores indicate higher burnout. This approach allows a more comprehensive examination of how POQ and LF-DJA are associated with burnout and its distinct dimensions.

Conservation of resources theory (COR), proposed by Hobfoll (1989), provides a resource-dynamics account of stress and coping. COR proposes that people seek to acquire, protect, and maintain valued resources and that stress is associated with actual resource loss, threatened loss, or insufficient resource gain after investment (Hobfoll, 2001). In work settings, employees who appraise their abilities as underused or their contributions as insufficiently returned may also report cognitive and emotional regulation costs. This theoretical logic supports an association-based account of resource threat without establishing the temporal direction of the present variables.

COR therefore helps interpret why POQ and LF-DJA may co-occur with burnout. POQ can reflect an appraisal that capability is not being activated; LF-DJA can reflect an appraisal that valued inputs are insufficiently returned. Together, these appraisals are theoretically consistent with resource threat and resource-depletion experiences that may be associated with emotional exhaustion and cynicism. The present design estimates this pattern statistically rather than observing a sequence of resource loss over time.

Social Exchange Theory (SET) was originally proposed by Homans (1958) and later systematized by Blau (1964). It has become a major theoretical framework for explaining interpersonal and organization–employee relationships in sociology and organizational behavior. SET posits that the relationship between employees and organizations is based on reciprocity, mutual obligation, and exchange.

In organizational contexts, employees invest not only time and labor but also knowledge, skills, experience, loyalty, and emotional resources. In return, they expect organizations to provide pay, recognition, developmental opportunities, respect, and other valued outcomes. When employees perceive that the organization does not respond to their contributions with commensurate returns, they may experience exchange imbalance, which can further trigger unfairness perceptions, reduced trust, and negative emotional responses (Cropanzano and Mitchell, 2005; Thau et al., 2007).

From a social-exchange perspective, employees with POQ may appraise their human-capital inputs as exceeding the demands of their job and may also appraise received outcomes as insufficiently commensurate with those inputs. This proposition is consistent with an association between POQ and LF-DJA, as well as an association between LF-DJA and workplace well-being. It does not establish that either appraisal temporally precedes the other. Together, SET and COR provide complementary interpretive lenses: SET clarifies why input-outcome appraisal is relevant, and COR clarifies why resource threat may co-occur with strain.

POQ reflects a subjective discrepancy between employees’ perceived capability supply and the challenge or use afforded by their job (Maynard et al., 2006). When employees appraise their knowledge, experience, and abilities as underused, they may also report value underrecognition, frustration, and strain (Bochoridou and Gkorezis, 2024; Erdogan and Bauer, 2009; Maynard and Parfyonova, 2013). These appraisals are theoretically consistent with emotional exhaustion and cynical detachment, but the present study evaluates their statistical association rather than a developmental sequence.

The Chinese public-sector sample provides a contextual backdrop in which high entry credentials can coexist with roles that employees may experience as routine or limited in immediate challenge. Such conditions may make capability underuse salient to employees, but the study does not measure role rules, promotion criteria, or institutional allocation processes. Any contextual description is therefore used only to situate employee appraisals of capability use, resource threat, and workplace well-being.

From the COR perspective, POQ represents inefficient resource utilization and insufficient return on resource investment (Cheng et al., 2021). Although employees possess valuable qualifications, these resources may not be effectively activated in their jobs. Instead, employees may be repeatedly exposed to work situations characterized by low challenge, poor fit, and limited meaning, which can be associated with sustained psychological strain (Sánchez-Cardona et al., 2020). When such strain is reported, employees may also report emotional exhaustion. They may also protect themselves by reducing emotional involvement and maintaining psychological distance from their work, which is consistent with stronger cynicism (Maslach and Leiter, 2016).

At the employee-appraisal level, POQ may co-occur with a sense that high human-capital investment has not been visibly used, recognized, or developed. Such an appraisal can be associated with lower emotional investment and greater psychological distance from work (Maslach and Leiter, 2016). The key point is not that a particular sectoral arrangement causes burnout, but that capability underuse may be experienced as resource threat and may covary with exhaustion and cynicism.

Based on this reasoning, POQ is hypothesized to be positively associated with emotional exhaustion and cynicism. Overall burnout is examined as an auxiliary summary. The POQ–LPE association is exploratory and non-directional because employees who regard themselves as overqualified may preserve strong self-evaluations of competence even when they report strain.

Within this framework, COR motivates the resource-underutilization account and SET specifies the exchange-appraisal component. JD-R situates these employee-level associations in the broader work-and-well-being literature. POQ remains a subjective capability-role appraisal rather than an objectively measured job demand.

H1: POQ is positively associated with emotional exhaustion and cynicism.

### 2.3 Less favorable distributive-justice appraisal as a proximal exchange evaluation

In this study, a less favorable distributive-justice appraisal (LF-DJA) denotes employees’ reverse-scored appraisal of whether outcomes such as pay, recognition, promotion opportunities, and developmental resources are commensurate with their contributions. The four items come from the distributive-justice dimension of Colquitt’s (2001) organizational-justice scale. Higher LF-DJA values indicate a less favorable appraisal of outcome fairness; they do not represent an independently measured or objectively verified circumstance of unfair outcome allocation.

Organizational-justice research differentiates outcome appraisal from procedural and interpersonal appraisal. The present measure is restricted to the distributive-justice items and therefore captures how employees evaluate outcomes relative to their inputs. It cannot determine whether a participating organization’s outcomes were objectively inequitable, whether procedures were fair, or whether interpersonal treatment shaped the response. This measurement boundary is central to the interpretation of the results.

The reverse-scored operationalization is also important conceptually. It is more precise to describe the variable as a less favorable distributive-justice appraisal than as a separate injustice construct. The analysis therefore distinguishes POQ, which concerns perceived capability-role mismatch, from LF-DJA, which concerns employees’ appraisal of outcome fairness. The two constructs can be closely related without being identical in definition or measurement.

LF-DJA is relevant to the present study because POQ may be especially salient when employees appraise work outcomes, recognition, or development as insufficiently commensurate with their inputs. This theoretical ordering fits social-exchange and COR reasoning, but it is not a temporal sequence observed in the present data. The empirical question is whether LF-DJA statistically accounts for an indirect association between POQ and burnout outcomes, especially emotional exhaustion and cynicism.

POQ need not show the same association with burnout for every employee. Negative reactions may be more likely when employees appraise their capability-role mismatch as accompanied by insufficient recognition, rewards, or opportunities. Such appraisals can be examined as correlates of resource threat and workplace well-being without claiming that the appraisal is a temporally verified causal mechanism.

SET provides a rationale for the hypothesized statistical ordering between POQ and LF-DJA. Employees who appraise their knowledge, skills, and experience as exceeding role requirements may also appraise received outcomes as insufficiently commensurate with their inputs (Cheng et al., 2020; Creed and Blume, 2013; Liu et al., 2015). COR provides a complementary rationale for why a less favorable outcome appraisal may co-occur with strain. Together, the theories support the indirect-association tests while leaving causal direction open.

In the sampled public-sector work settings, formalized roles and career structures may provide a backdrop in which employees become attentive to the fit between their contributions, opportunities, and recognition. These conditions are not measured in this study and are not used to infer a public-sector governance effect. They are relevant only as context for employees’ appraisals of capability use, resource threat, and workplace well-being.

COR theory also clarifies why LF-DJA may be associated with burnout. A less favorable outcome appraisal can signal to employees that invested resources have not yielded commensurate return or that future resource recovery is uncertain. Employees may then report disappointment, lower emotional investment, and greater detachment from work. This is a theoretical interpretation of observed associations, not evidence that the appraisal temporally produces burnout.

Integrating SET and COR, POQ can be treated as a capability-role appraisal and LF-DJA as an outcome-fairness appraisal. The theoretically ordered POQ-LF-DJA-burnout configuration is evaluated as an indirect association model. It does not establish an intervening mechanism, determine causal direction, or exclude the possibility that burnout or unobserved work characteristics influence both appraisals.

Burnout dimensions should not be assumed to follow a single pattern. Emotional exhaustion and cynicism are the primary outcomes because they map directly onto resource depletion and psychological withdrawal. LPE is retained as an exploratory competence-related outcome. Employees who appraise themselves as overqualified may report exhaustion and cynicism while maintaining a comparatively favorable view of their professional capability; any LF-DJA-related indirect association involving LPE is therefore examined without a directional expectation.

The appraisal focus also imposes an interpretive boundary. Employees may form a less favorable distributive-justice appraisal for reasons that are not identical to POQ, including observed comparison targets, pay policies, promotion timing, perceived procedural constraints, or interpersonal experiences. The present analysis does not assume that all such appraisals reflect objectively unfair distribution. It tests whether POQ covaries with this employee-reported appraisal and whether the appraisal is statistically linked with burnout. This distinction is particularly important in public-sector settings, where formal distribution rules may be stable and legitimate while their consequences may nevertheless be experienced as misaligned with individual capability, contribution, or development expectations.

Conceptually, this distinction positions distributive-justice appraisal as a proximal employee evaluation within the broader POQ-burnout pattern. POQ expresses an appraisal of capability-role mismatch; distributive-justice appraisal expresses a more focused judgment about the adequacy of received outcomes in relation to contribution. This sequence is theoretically consistent with social exchange and COR reasoning, but it remains a proposed interpretation rather than a temporal fact established by the present data. It is possible, for example, that employees with burnout symptoms appraise work conditions more negatively, or that unobserved features of jobs simultaneously influence POQ, fairness appraisal, and burnout. The analyses therefore estimate a theoretically informed conditional association model and locate claims about process within those design limits.

H2: A less favorable distributive-justice appraisal is positively associated with emotional exhaustion and cynicism.

H3: POQ is positively associated with a less favorable distributive-justice appraisal.

H4: LF-DJA statistically accounts for positive indirect associations between POQ and emotional exhaustion and cynicism.

The distinction between an LF-DJA and an objective distribution is important for the present design. The four Colquitt items were reverse-scored so that higher LF-DJA scores denote a less favorable appraisal of distributive fairness. Such scores may reflect employees’ evaluation of reward adequacy in relation to their own inputs, but they cannot determine whether organizational outcomes were objectively inequitable or whether other justice dimensions shaped the appraisal. The study therefore uses LF-DJA as a clearly defined employee-reported appraisal variable and interprets its statistical role within that measurement boundary.

### 2.4 Psychological capital as a conditional personal resource

Psychological capital (PsyCap) refers to a positive psychological resource configuration comprising hope, efficacy, resilience, and optimism (Luthans et al., 2007). In the present study, PsyCap is modeled as a second-order construct. Its score is used in interaction models to examine whether the magnitudes of the POQ-LF-DJA, POQ-burnout, and LF-DJA-burnout associations differ across levels of PsyCap. This approach evaluates conditional association, not a causal intervention effect.

COR theory suggests that employees with stronger personal resources may appraise resource threat differently and may report less strain under adverse work conditions. Employees higher in PsyCap may be more likely to use positive reappraisal, goal adjustment, and problem solving when they perceive capability underuse or less favorable outcomes (Pang et al., 2021; Zhang et al., 2010). The empirical question is therefore whether observed association magnitudes differ by PsyCap, not whether PsyCap eliminates workplace conditions that employees find constraining.

The sampled public-sector context does not change this employee-level interpretation. PsyCap is not presented as a response to, or substitute for, administrative reform. It is an individual resource that may condition how employees appraise capability use and outcome fairness in their everyday work, with implications for workplace well-being.

From a social-exchange perspective, employees with higher PsyCap may be less likely to make a globally negative appraisal of capability-role mismatch or outcome fairness. They may be more likely to seek feedback, reframe goals, or identify usable resources. These possibilities motivate tests of conditional associations but do not establish a behavioral sequence or determine whether the organization’s outcomes were objectively fair.

Taken together, the study tests whether PsyCap conditions associations among POQ, LF-DJA, and burnout outcomes. For LPE, the role of self-efficacy and hope may be particularly relevant because employees can preserve competence beliefs even when they report emotional strain. The hypotheses therefore differentiate the primary exhaustion and cynicism outcomes from the exploratory LPE outcome.

Although PsyCap is modeled as a higher-order personal resource, its inclusion does not imply that employees should adjust to structurally deficient work systems. PsyCap can shape appraisal and coping but cannot guarantee greater task challenge, more equitable advancement, or distributive outcomes that employees regard as commensurate with their contributions. Accordingly, the moderation hypotheses are framed as conditions under which observed associations may be weaker, rather than as an intervention model or a recommendation to individualize responsibility for burnout.

Treating PsyCap as a higher-order resource is appropriate to the present question because hope, efficacy, resilience, and optimism are expected to work jointly rather than as isolated moderators. Hope may support continued goal pursuit and flexible pathway generation; efficacy may sustain confidence when roles offer limited immediate challenge; resilience may facilitate recovery from disappointment; and optimism may prevent a local work setback from becoming a global expectation of resource loss. At the same time, this integrated measurement approach means that the analysis does not identify which of the four components is decisive. Future research could compare the higher-order resource model with component-specific mechanisms and examine whether self-efficacy is especially relevant to the competence-related LPE outcome.

H5: PsyCap conditions the POQ–LF-DJA association such that the positive association is weaker at higher levels of PsyCap.

H6: PsyCap conditions the POQ–burnout and LF-DJA–burnout associations, with weaker positive associations at higher PsyCap; emotional exhaustion and cynicism are evaluated separately.

Conditional indirect associations are examined as complementary extensions of these moderation hypotheses. Overall burnout remains auxiliary, and all LPE associations remain exploratory. The consolidation of hypotheses was undertaken during revision to distinguish the primary questions from auxiliary and exploratory analyses. The Results map the original H1–H8 labels and their subhypotheses to the revised presentation, including unsupported and specification-dependent findings.

The proposed role of PsyCap is conditional rather than compensatory. Higher PsyCap may be associated with a weaker POQ-LF-DJA association and with lower vulnerability to associated strain. Neither the construct nor the interaction tests imply that employees should accommodate persistent capability underuse or opaque recognition. Personal-resource development may complement, but cannot replace, work conditions that support meaningful capability use, transparent recognition, and employee well-being.

Figure 1 summarizes the hypothesized statistical ordering. Emotional exhaustion and cynicism are parallel primary outcomes, LPE is an exploratory parallel outcome, and overall burnout is an auxiliary summary. PsyCap enters the core structural equations as a personal-resource covariate; its interaction terms are evaluated in complementary models. Arrows indicate theoretical statistical ordering rather than an observed temporal sequence.

**Figure 1:**
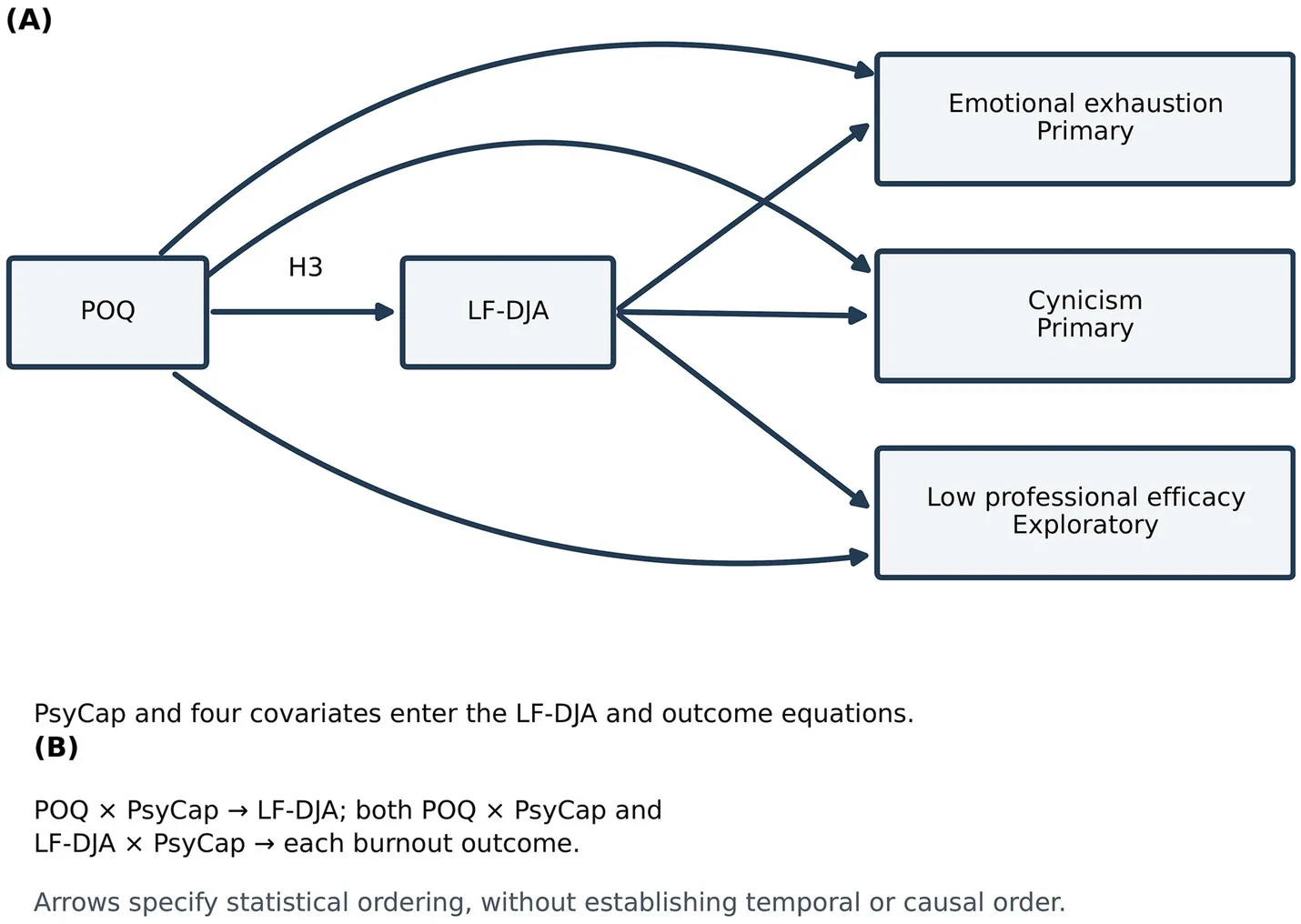
Revised conceptual statistical-ordering model. **(A)** Core statistical ordering. **(B)** Complementary moderation. PsyCap and the four covariates enter the LF-DJA and outcome equations. Arrows indicate statistical ordering without establishing temporal or causal order.

## 3 Materials and methods

### 3.1 Study design, participants, and procedure

Guided by the employee-level framework, the study examined reports of POQ, LF-DJA, PsyCap, and burnout outcomes. To reduce item-context effects and respondent burden, the study used a two-section completion design with partial and instructed temporal separation (Weng and McElroy, 2012). Data collection proceeded on a rolling basis from March 17 to April 20, 2025, with each participating organization using its own 14-day field window. The first section included demographic characteristics, control variables, and PsyCap; the later section included POQ, distributive-justice items, and burnout. Participants were instructed to complete the sections in sequence, typically 2–12 days apart. This separation distinguished PsyCap from the later-section variables but did not separate POQ, LF-DJA, and burnout from one another.

Participants were recruited from 21 organizations: seven universities, five state-owned enterprises, four hospitals, and five government agencies. Organizations were approached through the authors’ existing personal contacts and referrals, without formal organization-level eligibility criteria. The employee-level eligibility condition was current formal employment in a participating organization; no additional age, education, tenure, or rank restrictions were imposed. Employees were invited primarily through existing personal contacts and participated voluntarily. Managers were not informed of individual participation decisions and did not have access to individual responses.

The organizations were located primarily in Dandong and Shenyang, Liaoning Province, with smaller representation from Benxi and Beijing. The seven universities covered these four cities; the state-owned enterprises, hospitals, and government agencies were located in Dandong and Shenyang. This was a non-probability, multi-organization convenience field sample with limited national geographic coverage.

Two distribution procedures were used. In the batch procedure, 336 complete questionnaire sets were distributed through organizational contacts and 288 were returned. The procedure used anonymous matching codes and written instructions about completion order and timing; however, it did not generate participant-level timestamps. Consequently, the individual interval and compliance cannot be independently verified for this subgroup. This portion of the sample is therefore interpreted as having instructed, rather than verified, temporal separation. In the tracked procedure, 179 participants completed the first section in person at T1 and 174 matched participants completed the second section at T2 using the same non-identifying code. The code was used only for matching. No names, contact information, or other direct identifiers were collected, and organizational representatives did not have access to individual responses. In the tracked procedure, respondents received a small low-value token of appreciation that was not contingent on response content.

Of 462 returned questionnaires, 47 were excluded: 16 for straight-line responding, 14 for deterministic patterned responding, 12 for substantial missingness, and five for excessive endpoint responding. Each questionnaire was assigned one first-exclusion category. The final analytic sample included 415 employees: 182 university faculty or administrative staff, 98 state-owned-enterprise employees, 74 healthcare workers, and 61 government employees. The sample comprised 259 batch and 156 tracked respondents across 21 organizations, each contributing 9–34 participants (Table 1). All participants received an informed-consent statement explaining the study purpose, confidentiality safeguards, voluntary participation, and the right to withdraw without consequences. The study was reviewed and approved by the Ethics Committee of Northeastern University, China (Protocol Code: NEU-BA-202503-006; Approval Date: 5 March 2025), before data collection.

**Table 1:** Characteristics of the analytic sample.

Variable	Category	Frequency	Percentage
Gender	Male	178	42.89%
Gender	Female	237	57.11%
Age	Under 25 years	65	15.66%
Age	26–35 years	203	48.92%
Age	36–45 years	108	26.02%
Age	46 years and above	39	9.40%
Education	Junior college	0	0.00%
Education	Bachelor’s degree	107	25.78%
Education	Master’s degree	241	58.07%
Education	Doctoral degree	67	16.15%
Work tenure	Less than 1 year	59	14.22%
Work tenure	1–3 years	97	23.37%
Work tenure	4–6 years	147	35.42%
Work tenure	7–9 years	69	16.63%
Work tenure	More than 10 years	43	10.36%
Occupation type	University faculty and administrative staff	182	43.86%
Occupation type	State-owned enterprise employees	98	23.61%
Occupation type	Healthcare workers	74	17.83%
Occupation type	Government employees	61	14.70%
Collection procedure	Batch	259	62.41%
Collection procedure	Tracked	156	37.59%

*N* = 415. The sample covers 21 organizations, with 9–34 respondents per organization. Percentages may not sum to 100 because of rounding.

Questionnaires were excluded when they met the study’s data-quality screening criteria. Substantial missingness was defined as missing responses on at least 19 of the 54 focal measurement items (≥35% of the measurement battery). Excessive endpoint responding was defined as responding with either endpoint of the 7-point scale (1 or 7) on at least 39 of the 54 measurement items (≥72.2%), after excluding cases already identified as straight-line or deterministic patterned responding. Exclusion categories were applied as mutually exclusive first-exclusion classifications to avoid double counting. In the screening workbook, the straight-line category comprised records with the same stored value on all 54 focal items. Supplementary Tables S22, S23 report the screening criteria and case-level audit. All 415 retained cases had complete focal-item data.

During revision, the patterned-response classifications were audited in stored workbook column order: POQ (9 items), LF-DJA (4), EE (5), CY (4), professional efficacy (6), and the four PsyCap dimensions (7, 7, 6, and 6). This is the audit order, not the order of questionnaire administration. Four templates exactly matched the first 22 and final 26 stored items, restarting at the beginning of each segment: an ascending 1–7 cycle (four records), a descending 7–1 cycle (four), an alternating 1–7 sequence (three), and a repeating 2–2–6–6 sequence (three). The corresponding six-item professional-efficacy sequences were exactly 3–4–2–3–4–2, 3–2–4–3–2–4, 2–4–2–4–2–4, and 4–4–2–2–4–4, respectively. The last sequence is a six-item prefix of a period-four sequence, not two complete repetitions. These patterns describe the stored screening records; they should not be equated with sequences of raw response options without reference to the original scoring records.

The inclusion of several public-sector organization types provided occupational-context heterogeneity, rather than a representative estimate of the Chinese public sector. The substantive analyses were conducted at the employee level and controlled for gender, age, education, and tenure. Procedure, occupational context, and organization membership were examined in sensitivity analyses; these analyses do not identify institutional causal effects.

### 3.2 Measures

All focal constructs were measured using established instruments, and the questionnaire was administered in Chinese. PsyCap used the Chinese-language version reported by Zhang et al. (2010). Unless otherwise specified, responses used a 7-point Likert scale ranging from 1 (strongly disagree) to 7 (strongly agree). Measure-specific scoring and language adaptation are described below.

For POQ and distributive justice, the original English instruments were used as source versions during questionnaire development as references for the intended item content and construct definitions, and study-specific Chinese versions were prepared using a forward–back translation procedure. For burnout, the administered 15-item configuration followed the Chinese MBI-GS revision reported by Li and Shi (2003), with the original English MBI-GS retained as a reference for item content and construct meaning during questionnaire preparation. This distinction clarifies the source of the 15-item configuration without implying that previously published Chinese adaptations were invalid.

Perceived overqualification. POQ was measured with the nine-item unidimensional scale developed by Maynard et al. (2006). The original English-language items were the source for the study-specific Chinese translation. The scale assesses the extent to which respondents believe that their education, experience, and skills exceed the requirements of their job. Higher scores indicate stronger POQ. Cronbach’s alpha was 0.90.

Less favorable distributive-justice appraisal. Four distributive-justice items from Colquitt’s (2001) original English-language organizational-justice scale were translated into Chinese and reverse-scored for analysis. Higher values therefore indicate a less favorable distributive-justice appraisal regarding outcomes such as pay, recognition, rewards, promotion opportunities, and developmental resources in relation to one’s contributions. Cronbach’s alpha was 0.86. This operationalization is used consistently throughout the paper; it should not be interpreted as a direct, independent measure of objective unfairness.

Burnout. Job burnout was assessed using a 15-item MBI-GS configuration based on the Chinese revision reported by Li and Shi (2003), comprising five emotional-exhaustion items, four cynicism items, and six professional-efficacy items. Li and Shi’s revision was developed from the original 16-item MBI-GS (Schaufeli et al., 1996); during their revision work, one cynicism item (Item 13 in the initial 16-item Chinese questionnaire) was removed because it showed substantial cross-loading in exploratory factor analysis. The resulting 5–4–6 configuration therefore predated the present study and was adopted during questionnaire development, before data collection or examination of the present results. The original English MBI-GS was retained as a reference for item content and construct meaning when the administered Chinese wording was checked. No burnout item was removed or selected on the basis of the current sample’s reliability, factor loadings, AVE, or SEM results. Professional efficacy was reverse-scored and labelled low professional efficacy (LPE), such that higher scores reflect lower professional efficacy. Cronbach’s alpha values were 0.84 for emotional exhaustion, 0.78 for cynicism, and 0.90 for LPE.

Psychological capital. PsyCap was measured using the 26-item Chinese Positive Psychological Capital Questionnaire (PPQ) reported by Zhang et al. (2010), comprising self-efficacy (seven items), resilience (seven), hope (six), and optimism (six). These items were not newly translated for this study. Higher scores indicate stronger PsyCap. Cronbach’s alpha values for the four first-order dimensions were 0.90, 0.91, 0.93, and 0.90, respectively. The SEM specified these four dimensions as indicators of a second-order PsyCap factor. In complementary observed-score analyses, PsyCap was the equally weighted mean of the four dimension means; the auxiliary overall-burnout summary was the equally weighted mean of EE, CY, and LPE scores.

For POQ and distributive justice, bilingual researchers translated the original English wording into Chinese, an independent bilingual reviewer back-translated the items, and discrepancies were resolved by comparison with the intended construct definitions. For burnout, the 15-item configuration was based on Li and Shi’s (2003) Chinese MBI-GS revision, while the original English MBI-GS was used as a reference when checking item content, Chinese wording, and construct meaning. Contextual wording changes were limited to clarity for the sampled work settings. PsyCap was excluded from this translation procedure because the Chinese-language PPQ was used. These source and language-preparation distinctions are reported to clarify provenance; the present sample’s reliability and validity checks do not establish cross-cultural measurement equivalence.

Control variables. Gender, age, education, and work tenure were included as covariates because these characteristics are commonly related to burnout and work experiences (Farshi and Omranzadeh, 2014; Stanetić and Tesanović, 2013).

### 3.3 Analytic strategy and data-quality checks

The primary analysis was an item-level latent-variable structural equation model of the core direct and indirect associations. POQ, LF-DJA, emotional exhaustion, cynicism, and LPE were specified as distinct first-order factors. The four PsyCap dimensions were modeled as first-order factors loading on a second-order PsyCap factor. All 54 measurement items were retained. EE and CY were primary outcomes; LPE was exploratory. Overall burnout was analyzed separately as an auxiliary composite and was not entered alongside its components as another latent outcome.

The structural equations regressed LF-DJA on POQ, PsyCap, and the four covariates, and each burnout dimension on POQ, LF-DJA, PsyCap, and the same covariates. Exogenous variables covaried freely, and the three outcome disturbances were allowed to covary. Item-error covariances and residual covariances among the first-order PsyCap dimensions were fixed to zero. Marker indicators fixed the first loading of each first-order factor to one; the self-efficacy loading on second-order PsyCap was also fixed to one. Covariates retained their original numerical coding. The covariance model contained 58 observed variables and 161 free parameters.

Normal-theory maximum likelihood was used for covariance estimation, treating item responses as continuous. Direct-path inference used observed-information sandwich standard errors and two-sided Wald tests. Indirect and total associations used percentile 95% intervals from 5,000 case-resampled bootstrap fits, with the same model refitted in each sample. All 5,000 fits converged. Conventional, unscaled ML fit indices are reported. The core structural component is saturated conditional on the measurement structure and covariates; fit therefore assesses the measurement restrictions and does not identify the direction of the POQ–LF-DJA–burnout ordering. Additional measurement specifications and loadings are provided in Supplementary Tables S24, S25.

Complementary observed-score interaction models used mean-centered POQ, LF-DJA, and PsyCap. The LF-DJA equation included POQ, PsyCap, POQ × PsyCap, and the four covariates. Each outcome equation included POQ, LF-DJA, PsyCap, POQ × PsyCap, LF-DJA × PsyCap, and the same covariates. This unified specification estimates both outcome interactions together, rather than combining coefficients from separate models. Conditional indirect associations were computed as the product of the conditional POQ–LF-DJA and LF-DJA–outcome coefficients at mean PsyCap and at one SD below and above the mean; high-minus-low differences were bootstrapped with 5,000 shared case resamples.

PROCESS Models 1, 4, 7, 14, and 58 provide complementary specification comparisons in the Supplementary material. Supplementary Table S20 reports Model 14 moderated-mediation indices, defined as the POQ–LF-DJA coefficient multiplied by the LF-DJA × PsyCap coefficient, with bootstrap SEs and intervals. This statistical index does not establish causal mediation. The restricted Model 58 comparison in Supplementary Table S21 was re-estimated using the same 415 cases, gender, age, education, tenure, score construction, centering, PsyCap levels, and 5,000 case resamples as the unified models. Both equations include the four covariates. The LF-DJA equation is identical to the unified specification; only POQ × PsyCap is omitted from the outcome equation. Original analyses used SPSS 24.0, AMOS 24.0, SmartPLS 4.0, and PROCESS 4.2. The additional SEM, bootstrap, regression, and sensitivity computations were implemented in Python 3.12 with NumPy 2.3 and SciPy 1.17. These revision-stage analyses were not preregistered.

Procedure sensitivity analyses added a batch/tracked indicator and tested procedure interactions with the focal predictors. Occupational-context sensitivity analyses added indicators for state-owned enterprise, healthcare, and government employment, using university employment as the reference. Organization membership was assessed using random-intercept ICC estimates and bias-reduced cluster-robust regression standard errors (CR2) with coefficient-specific Satterthwaite degrees of freedom. Given 21 organizations, this small-sample adjustment was preferred to unadjusted large-sample cluster inference (Pustejovsky and Tipton, 2018). These observed-score analyses assess sensitivity to sample context and dependence; they do not constitute multigroup latent measurement-invariance tests. Detailed procedure, occupational-context, and cluster results are provided in Supplementary Tables S26, S27. An additional sensitivity check reinstated the five complete records excluded only for endpoint responding (*N* = 420). The unified observed-score equations were refitted using the same score construction and four covariates, with focal predictors centered within the expanded sample. Inference used conventional OLS t tests. Conditional indirect associations and high-minus-low differences used 5,000 shared case resamples with percentile 95% intervals and seed 20,260,922, evaluated at the expanded sample’s mean and ±1 SD of PsyCap. Supplementary Section 4.9 and Supplementary Tables S29–S31 document the reinstated records, refitted coefficients, and bootstrap estimates.

An *a priori* power calculation was not documented. A revision-stage non-central-F sensitivity calculation for one incremental interaction term, *N* = 415, α = 0.05, and nine predictors in the full outcome equation indicated minimum detectable f^2^ values of 0.0190 at 80% power and 0.0254 at 90% power. These calculations assume independent observations and concern a single regression interaction, not the power of the full SEM or conditional indirect association. Smaller interactions may remain undetected, and inference emphasizes interval width rather than observed post-hoc power.

All significance tests were two-sided with α = 0.05. No formal multiplicity correction was imposed. Interpretation considered effect magnitude, confidence intervals, the distinction among primary, auxiliary, and exploratory outcomes, and consistency across specifications. Borderline *p* values were not treated as strong evidence, and *p* < 0.10 was not used as a separate significance category. A non-significant coefficient was interpreted as insufficient evidence, not as evidence of a zero association or equivalence.

Potential nonresponse bias was assessed through early-late respondent comparisons (Armstrong and Overton, 1977). These comparisons used return order, not the batch/tracked procedure labels, and cannot establish absence of nonresponse or procedure-related bias (Supplementary Tables S1, S2). Common-method concerns were addressed through section separation, anonymity, voluntary participation, and statistical diagnostics. Harman’s single-factor test indicated that the first unrotated factor explained 31.55% of variance, below the commonly used 40% reference, and the one-factor CFA model fit substantially worse than the focal measurement model (Supplementary Tables S3, S4). These checks do not rule out self-report bias; they indicate only that single-factor common-method variance did not appear dominant.

Multicollinearity diagnostics for all predictors in the unified outcome equations are reported in Supplementary Table S5. The largest VIFs were 5.83 for age and 6.09 for tenure, consistent with their strong correlation. Both covariates were retained to preserve the original adjustment set; their shared variation warrants caution in interpreting their individual coefficients.

The measurement evaluation followed several complementary checks. Cronbach’s alpha and composite reliability were examined to assess internal consistency. Average variance extracted (AVE) and standardized factor loadings were used to examine convergent validity. The square roots of AVE and heterotrait-monotrait ratios were examined as discriminant-validity diagnostics. PsyCap was evaluated through its four first-order dimensions and its higher-order structure; the new SEM specifically used a reflective second-order factor. Detailed loadings, AVE, composite-reliability, HTMT point estimates, and HTMT bootstrap confidence intervals are reported in Supplementary Tables S6–S10. These diagnostics inform interpretation of the conditional-process analyses; they do not establish uniformly adequate convergent validity across specifications.

The initial CFA checks distinguished POQ, LF-DJA, the auxiliary overall-burnout representation, and PsyCap and compared less differentiated alternatives (Supplementary Table S4). The new core SEM instead separates EE, CY, and LPE at the item level, so its fit and paths are reported separately below. Neither set of results eliminates the substantial POQ–LF-DJA association or establishes a temporal sequence.

## 4 Results

### 4.1 Measurement quality and descriptive statistics

The initial auxiliary-outcome CFA provided acceptable support for distinguishing POQ, LF-DJA, the auxiliary overall-burnout summary, and PsyCap: χ^2^(1,301) = 2,162.41, χ^2^/df = 1.66, RMSEA = 0.04, CFI = 0.94, TLI = 0.93, IFI = 0.94, and SRMR = 0.05. All six three-factor alternatives, the three two-factor alternatives, and the one-factor alternative fit less well (CFI = 0.54–0.74; RMSEA = 0.08–0.11; Supplementary Table S4). For the initial all-item PsyCap measurement checks, Cronbach’s alpha was 0.96, composite reliability was 0.96, and average variance extracted was 0.52 (Supplementary Table S6). These values do not characterize the second-order factor in the revised core SEM.

The measurement evidence defines a necessary boundary for interpreting LF-DJA. The reverse-scored distributive-justice items capture employees’ outcome-fairness appraisal; they do not verify that rewards, promotion, or allocation decisions were objectively unjust. The CFA therefore supports differentiated employee-report constructs, not an audit of organizational fairness.

The initial measurement checks and the revised core SEM are reported separately. In the revised core SEM, standardized item loadings ranged from 0.630 to 0.853 (Supplementary Table S25). For CY, AVE calculated from the reported standardized loadings was approximately 0.473, below the conventional 0.50 reference, despite acceptable internal consistency (Cronbach’s alpha = 0.78). CY is retained with this convergent-validity limitation. The initial all-item PsyCap CR of 0.96 and AVE of 0.52 do not describe the second-order factor in the revised SEM. Detailed loadings, AVE, composite-reliability, HTMT point estimates, and HTMT bootstrap confidence intervals are reported in Supplementary Tables S6–S10.

Table 2 presents descriptive statistics, internal consistency, and correlations for the primary constructs and separate burnout dimensions. POQ was strongly correlated with LF-DJA (r = 0.74), while its correlations with EE and CY were approximately 0.12. LF-DJA correlated approximately 0.19 with EE and 0.17 with CY. These small zero-order burnout associations should be considered alongside adjusted coefficients and their confidence intervals.

**Table 2:** Descriptive statistics and correlations for the focal constructs and burnout dimensions.

Variable	Mean	SD	α	1	2	3	4	5	6
1. POQ	4.37	1.31	0.90	—					
2. LF-DJA	4.14	1.48	0.86	0.74	—				
3. PsyCap	4.14	1.14	0.96	0.54	0.54	—			
4. EE	3.81	1.43	0.84	0.12	0.19	0.00	—		
5. CY	3.63	1.35	0.78	0.12	0.17	−0.02	0.68	—	
6. LPE	3.01	0.40	0.90	−0.18	−0.14	−0.22	0.01	0.02	—

*N* = 415. α = Cronbach’s alpha. Values below the diagonal are Pearson correlations. EE and CY are primary outcomes; LPE is exploratory. Overall burnout is an auxiliary summary and is not combined with its component dimensions in the primary latent model.

At the burnout-dimension level, POQ and LF-DJA were positively associated with emotional exhaustion and cynicism, the primary outcomes, but negatively associated with LPE (Table 2; Supplementary Table S8). This pattern indicates emotional and attitudinal strain without a parallel decline in competence-related self-evaluation. LPE is therefore interpreted as an exploratory outcome rather than evidence against the exhaustion and cynicism findings.

The POQ-LF-DJA correlation is substantial and is reported directly rather than minimized. The constructs nevertheless remain distinguishable: they have different construct definitions, the four-factor CFA showed acceptable fit (χ^2^/df = 1.66, CFI/TLI = 0.94/0.93, RMSEA = 0.04, SRMR = 0.05), their HTMT estimate was 0.83 with a 95% bootstrap confidence interval of [0.78, 0.87], and their square roots of AVE were 0.75 and 0.84 (Supplementary Tables S4, S8–S10). The HTMT point estimate is below the conservative 0.85 heuristic, although its upper confidence limit slightly exceeds that reference; the interval remains clearly below 1.00. Taken together, the evidence supports retaining POQ and LF-DJA as theoretically distinct but closely co-occurring employee appraisals, and it warrants cautious interpretation rather than a claim of complete empirical independence or temporal ordering.

This interpretation is more informative than treating the correlation as either a measurement failure or proof of a temporal mechanism. POQ answers whether employees regard their capability as exceeding role requirements; LF-DJA answers whether they appraise received outcomes as commensurate with contribution. The conditional association analyses retain both appraisals because each identifies a different employee-evaluation boundary relevant to workplace well-being.

Retaining both appraisals does not make LF-DJA an independent causal mechanism through which POQ produces burnout. Indirect-association estimates are a statistical decomposition conditional on the chosen model, and shared appraisal variance and unmeasured work conditions remain relevant to their interpretation.

### 4.2 Structural equation model of core associations

The core latent SEM yielded χ^2^(1,550) = 2,512.25, χ^2^/df = 1.62, CFI = 0.935, TLI = 0.931, RMSEA = 0.039 (90% CI [0.036, 0.041]), and SRMR = 0.046. All measurement-error and first-order PsyCap residual variances were positive. These conventional ML fit indices indicate an adequate approximation of the covariance structure under the specified measurement restrictions; they do not distinguish causal directions. Figure 2 presents the focal standardized paths from this single model, and Table 3 reports direct coefficients with robust standard errors.

**Figure 2:**
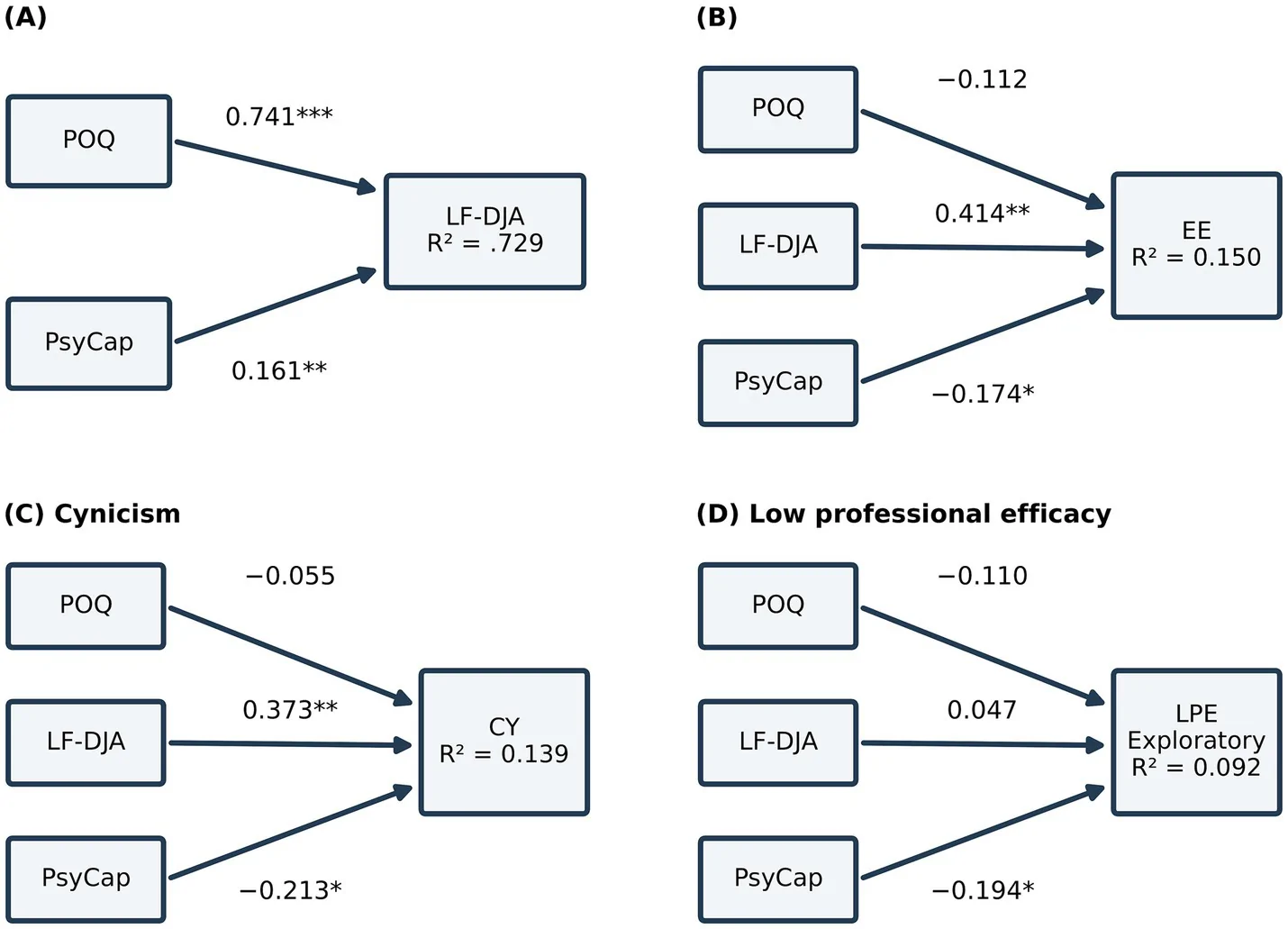
Focal standardized associations estimated in one latent structural equation model. **(A)** LF-DJA; **(B)** emotional exhaustion; **(C)** cynicism; **(D)** low professional efficacy. All four panels are from the same model. POQ, perceived overqualification; LF-DJA, less favorable distributive-justice appraisal; PsyCap, psychological capital; EE, emotional exhaustion; CY, cynicism; LPE, low professional efficacy. Covariate paths, measurement loadings, and residual covariances are omitted for readability. *p < 0.05, **p < 0.01, ***p < 0.001, using sandwich Wald tests. Unmarked paths are not statistically distinguishable from zero. Arrows do not establish temporal or causal direction. Overall burnout is retained only as a separate auxiliary observed-score analysis and is not an additional latent outcome.

**Table 3:** Direct associations in the core latent structural equation model.

Outcome	Predictor	B	β	Robust SE	95% CI	p
LF-DJA	POQ	0.742	0.741	0.064	[0.617, 0.867]	<0.001
LF-DJA	PsyCap	0.182	0.161	0.064	[0.056, 0.308]	0.005
EE	POQ	−0.119	−0.112	0.132	[−0.378, 0.141]	0.370
EE	LF-DJA	0.439	0.414	0.135	[0.175, 0.703]	0.001
EE	PsyCap	−0.209	−0.174	0.089	[−0.383, −0.035]	0.018
CY	POQ	−0.047	−0.055	0.120	[−0.281, 0.188]	0.696
CY	LF-DJA	0.319	0.373	0.123	[0.077, 0.560]	0.010
CY	PsyCap	−0.206	−0.213	0.082	[−0.366, −0.046]	0.012
LPE	POQ	−0.037	−0.110	0.046	[−0.126, 0.052]	0.417
LPE	LF-DJA	0.016	0.047	0.046	[−0.075, 0.107]	0.731
LPE	PsyCap	−0.074	−0.194	0.031	[−0.134, −0.014]	0.015

*N* = 415. B uses marker-indicator units; β is fully standardized. SEs, 95% CIs, and p values use observed-information sandwich inference. All equations adjust for gender, age, education, and tenure. The three outcome disturbances covary. R^2^: LF-DJA = 0.729, EE = 0.150, CY = 0.139, LPE = 0.092. No multiplicity correction was applied.

POQ was positively associated with LF-DJA (B = 0.742, β = 0.741, 95% CI [0.617, 0.867], *p* < 0.001). LF-DJA was positively associated with EE (B = 0.439, β = 0.414, 95% CI [0.175, 0.703], *p* = 0.001) and CY (B = 0.319, β = 0.373, 95% CI [0.077, 0.560], *p* = 0.010). After LF-DJA, PsyCap, and the covariates were included, the POQ direct associations were not statistically distinguishable from zero for EE (B = −0.119, *p* = 0.370), CY (B = −0.047, *p* = 0.696), or LPE (B = −0.037, *p* = 0.417). PsyCap was negatively associated with EE, CY, and LPE in this additive latent model. The explained variances were 0.729 for LF-DJA, 0.150 for EE, 0.139 for CY, and 0.092 for LPE.

The distinction between total and residual direct association is central to the interpretation. POQ had positive total associations with EE and CY, while its residual direct coefficients became small and imprecise after the closely related LF-DJA appraisal was included. This is compatible with the original positive-association hypotheses at the total-association level; it does not support independent positive direct POQ paths after simultaneous adjustment.

Percentile bootstrap intervals supported positive LF-DJA-related indirect associations with EE (B = 0.326, bootstrap SE = 0.110, 95% CI [0.127, 0.562]) and CY (B = 0.237, bootstrap SE = 0.100, 95% CI [0.060, 0.454]). The corresponding total associations were 0.207 (95% CI [0.049, 0.353]) and 0.190 (95% CI [0.050, 0.330]). The LPE indirect association was 0.012 (95% CI [−0.058, 0.084]), and its total association was −0.025 (95% CI [−0.078, 0.026]). Table 4 presents these estimates. The results support H2–H4 for EE/CY and H1 at the total-association level, while LPE remains exploratory.

**Table 4:** Indirect and total associations in the core latent model.

Outcome	Association	B	β	Boot SE	95% bootstrap CI
EE	Indirect	0.326	0.307	0.110	[0.127, 0.562]
CY	Indirect	0.237	0.277	0.100	[0.060, 0.454]
LPE	Indirect	0.012	0.035	0.036	[−0.058, 0.084]
EE	Total	0.207	0.195	0.077	[0.049, 0.353]
CY	Total	0.190	0.222	0.071	[0.050, 0.330]
LPE	Total	−0.025	−0.074	0.026	[−0.078, 0.026]

*N* = 415. Bootstrap SEs and percentile 95% CIs are based on 5,000 case-resampled fits; all fits converged. Indirect associations are specified through LF-DJA. B uses marker-indicator units and β is fully standardized. The same model and covariates as Table 3 apply. Arrow notation denotes statistical ordering rather than observed temporal sequence.

In the complementary additive observed-score specification, the auxiliary overall-burnout indirect association was 0.105 (95% CI [0.049, 0.164]) and its total association was 0.101 (95% CI [0.026, 0.170]). These estimates also controlled PsyCap and therefore differ from the original Model 4 specification without PsyCap. The auxiliary result is retained for completeness and is not treated as a substitute for the separate EE and CY findings.

Because POQ, LF-DJA, and burnout were measured together, neither the positive indirect estimates nor the change in the POQ coefficient identifies an intervening causal process or substantive suppression mechanism. The latent SEM accounts for specified measurement error but cannot remove confounding, establish temporal precedence, or make the two appraisals empirically independent.

### 4.3 Complementary psychological capital moderation analyses

Table 5 presents the unified observed-score equations. POQ × PsyCap was negative for LF-DJA (B = −0.342, SE = 0.033, *p* < 0.001), EE (B = −0.426, SE = 0.077, *p* < 0.001), and CY (B = −0.280, SE = 0.073, *p* < 0.001). LF-DJA × PsyCap was not statistically distinguishable from zero for EE (B = 0.040, *p* = 0.521) but was nominally significant for CY (B = −0.117, *p* = 0.0493). The CY result is close to the conventional threshold and warrants caution given multiplicity. For LPE, POQ × PsyCap yielded *p* = 0.0543 and LF-DJA × PsyCap *p* = 0.965; these results do not establish stable exploratory moderation. Table 6 summarizes the procedure, occupational-context, and organization-cluster sensitivity checks discussed below.

**Table 5:** Focal coefficients in the unified observed-score interaction models.

Outcome	Predictor	B	SE	95% CI	*p*
LF-DJA	POQ	0.500	0.043	[0.415, 0.585]	<0.001
LF-DJA	PsyCap	0.305	0.045	[0.217, 0.394]	<0.001
LF-DJA	POQ × PsyCap	−0.342	0.033	[−0.407, −0.276]	<0.001
EE	POQ	−0.058	0.078	[−0.212, 0.097]	0.462
EE	LF-DJA	−0.005	0.075	[−0.153, 0.144]	0.949
EE	PsyCap	−0.018	0.073	[−0.160, 0.125]	0.809
EE	POQ × PsyCap	−0.426	0.077	[−0.578, −0.275]	<0.001
EE	LF-DJA × PsyCap	0.040	0.063	[−0.083, 0.164]	0.521
CY	POQ	−0.090	0.074	[−0.236, 0.056]	0.226
CY	LF-DJA	0.001	0.071	[−0.139, 0.142]	0.984
CY	PsyCap	−0.075	0.069	[−0.210, 0.060]	0.273
CY	POQ × PsyCap	−0.280	0.073	[−0.423, −0.137]	<0.001
CY	LF-DJA × PsyCap	−0.117	0.059	[−0.234, −0.0004]	0.0493
LPE	POQ	−0.020	0.023	[−0.066, 0.026]	0.394
LPE	LF-DJA	0.027	0.022	[−0.017, 0.072]	0.222
LPE	PsyCap	−0.073	0.022	[−0.116, −0.031]	<0.001
LPE	POQ × PsyCap	0.044	0.023	[−0.001, 0.089]	0.0543
LPE	LF-DJA × PsyCap	−0.001	0.019	[−0.038, 0.036]	0.965
Overall burnout	POQ	−0.056	0.047	[−0.147, 0.036]	0.231
Overall burnout	LF-DJA	0.008	0.045	[−0.080, 0.096]	0.858
Overall burnout	PsyCap	−0.055	0.043	[−0.140, 0.029]	0.199
Overall burnout	POQ × PsyCap	−0.221	0.046	[−0.310, −0.131]	<0.001
Overall burnout	LF-DJA × PsyCap	−0.026	0.037	[−0.099, 0.047]	0.489

*N* = 415. B is unstandardized. LF-DJA is regressed on POQ, PsyCap, POQ × PsyCap, and four covariates; each outcome is regressed on POQ, LF-DJA, PsyCap, both product terms, and the same covariates. Continuous focal predictors are mean-centered. CIs and p values use conventional OLS t inference; cluster sensitivity is reported separately. R^2^: LF-DJA = 0.664, EE = 0.206, CY = 0.203, LPE = 0.099, overall burnout = 0.230. *p* values near 0.05 and CI limits that would round to zero are shown to four decimals; p values are unadjusted for multiplicity.

**Table 6:** Sensitivity of the focal observed-score coefficients.

Association	Base B	+ Procedure B	+ Sector B	+ Both B	CR2 SE	CR2 p
POQ → LF-DJA	0.705	0.705	0.702	0.702	0.040	<0.001
LF-DJA → EE	0.241	0.240	0.239	0.238	0.064	0.002
LF-DJA → CY	0.204	0.204	0.205	0.205	0.055	0.002
POQ × PsyCap → LF-DJA	−0.342	−0.342	−0.343	−0.344	0.026	<0.001
POQ × PsyCap → EE	−0.426	−0.428	−0.424	−0.426	0.059	<0.001
POQ × PsyCap → CY	−0.280	−0.282	−0.282	−0.283	0.067	<0.001
LF-DJA × PsyCap → EE	0.040	0.041	0.037	0.037	0.047	0.404
LF-DJA × PsyCap → CY	−0.117	−0.117	−0.115	−0.115	0.053	0.043

Each cell in the first four numerical columns is B from the same observed-score equation with the indicated additional covariates. Procedure adds tracked versus batch; sector adds three indicators with university as reference. CR2 SE and p apply to the baseline equation, clustered by 21 organizations, with Satterthwaite degrees of freedom. This table concerns regressions, not refits of the latent SEM.

The POQ–LF-DJA simple slope was 0.889 at low PsyCap (95% CI [0.806, 0.973]) and 0.111 at high PsyCap (95% CI [−0.026, 0.247]). For EE, the conditional POQ slopes were 0.428 (95% CI [0.184, 0.672]) and −0.544 (95% CI [−0.761, −0.326]); for CY, they were 0.229 (95% CI [−0.002, 0.460]) and −0.409 (95% CI [−0.615, −0.203]). Low and high denote one SD below and above mean PsyCap. Figure 3 shows predictions from these equations. The negative high-PsyCap slopes indicate a conditional sign difference over the plotted range, not that PsyCap universally lowers burnout or eliminates risk.

**Figure 3:**
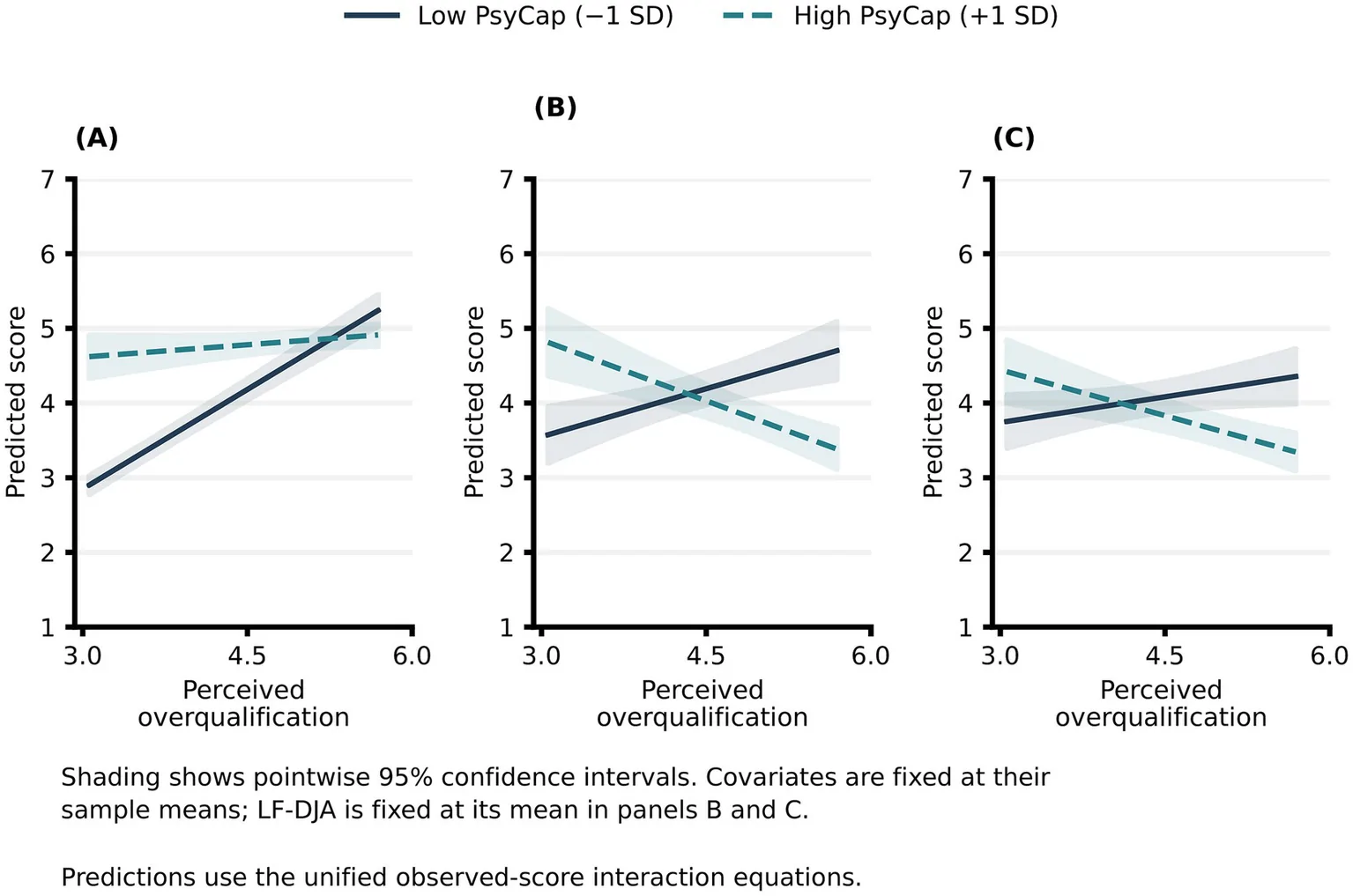
Conditional POQ associations with LF-DJA, emotional exhaustion, and cynicism in the unified interaction models. Low/high PsyCap are one SD below/above its mean. Shading shows pointwise 95% confidence intervals. Covariates are fixed at their sample means; LF-DJA is fixed at its mean in the EE and CY panels. These are observed-score predictions from the joint equations, not latent SEM coefficients. Higher LF-DJA indicates a less favorable distributive-justice appraisal. **(A)** LF-DJA; **(B)** emotional exhaustion; **(C)** cynicism.

For the auxiliary overall-burnout summary, POQ × PsyCap remained negative (B = −0.221, *p* < 0.001), whereas LF-DJA × PsyCap was not statistically distinguishable from zero (B = −0.026, *p* = 0.489). PsyCap retained a negative main association with LPE in the unified equation (B = −0.073, *p* = 0.001). Evidence therefore supports H5 and the POQ component of H6 for the primary outcomes; support for the LF-DJA component of H6 is limited to weak, outcome-specific CY evidence.

The separate PROCESS specifications provide additional conditional patterns (Supplementary Tables S17–S21). In the covariate-matched restricted Model 58 comparison, conditional indirect intervals excluded zero for EE at low and mean PsyCap and for CY at low PsyCap (Supplementary Table S21). These intervals included zero in the unified models (Table 7). Because the sample, covariates, scores, centering, moderator levels, and bootstrap resamples were held constant, this comparison isolates the inclusion of POQ × PsyCap in the outcome equation. The restricted estimates therefore indicate specification dependence rather than robustness of a conditional indirect pathway.

**Table 7:** Conditional indirect associations in the unified interaction specification.

Outcome	PsyCap level/contrast	Estimate	Boot SE	95% bootstrap CI
EE	Low (−1 SD)	−0.045	0.083	[−0.204, 0.119]
EE	Mean	−0.002	0.035	[−0.071, 0.067]
EE	High (+1 SD)	0.005	0.013	[−0.019, 0.035]
EE	High − low	0.050	0.083	[−0.115, 0.209]
CY	Low (−1 SD)	0.120	0.084	[−0.039, 0.293]
CY	Mean	0.001	0.036	[−0.069, 0.073]
CY	High (+1 SD)	−0.015	0.016	[−0.050, 0.011]
CY	High − low	−0.134	0.084	[−0.305, 0.026]
LPE	Low (−1 SD)	0.025	0.034	[−0.047, 0.089]
LPE	Mean	0.014	0.012	[−0.011, 0.037]
LPE	High (+1 SD)	0.003	0.004	[−0.004, 0.013]
LPE	High − low	−0.022	0.034	[−0.085, 0.048]
Overall burnout	Low (−1 SD)	0.033	0.049	[−0.060, 0.131]
Overall burnout	Mean	0.004	0.021	[−0.037, 0.047]
Overall burnout	High (+1 SD)	−0.002	0.008	[−0.020, 0.014]
Overall burnout	High − low	−0.036	0.048	[−0.132, 0.058]

*N* = 415. Estimates are the product of the two conditional component coefficients from the unified equations in Table 5. Low/mean/high PsyCap correspond to −1 SD/mean/+1 SD. Intervals are percentile bootstrap 95% CIs from 5,000 shared case resamples. Both outcome interactions are included; the covariate-matched restricted Model 58 comparison in Supplementary Table S21 omits POQ × PsyCap from the outcome equation. High − low is a direct bootstrap comparison. All intervals include zero.

In the unified specification, none of the conditional indirect-association intervals at low, mean, or high PsyCap excluded zero for EE, CY, LPE, or the auxiliary overall score (Table 7; Supplementary Table S28). For EE, the low- and high-PsyCap estimates were −0.045 (95% CI [−0.204, 0.119]) and 0.005 (95% CI [−0.019, 0.035]); for CY, they were 0.120 (95% CI [−0.039, 0.293]) and −0.015 (95% CI [−0.050, 0.011]). High-minus-low differences also included zero: 0.050 (95% CI [−0.115, 0.209]) for EE and −0.134 (95% CI [−0.305, 0.026]) for CY. Thus, the original H8 conditional-indirect pattern was not robust to the unified specification. This does not negate the positive indirect associations in the additive core SEM, which estimates a different model.

The clearest conditional finding is the negative POQ × PsyCap term in the LF-DJA equation. POQ-related outcome interactions also persisted, whereas LF-DJA-related moderation and conditional indirect associations depended on outcome and specification. These findings should not be reduced to a claim that PsyCap buffers every association. Table 8 preserves the mapping and evidential status of the original hypotheses.

**Table 8:** Traceability of the original hypotheses and revised evidence.

**Original hypothesis**	**Revised location**	**Evidence retained**
H1a/H1b	H1; Tables 3, 4	Positive total associations with EE/CY; residual direct POQ paths are not supported.
H2a/H2b	H2; Table 3	Positive LF-DJA paths to EE/CY in the additive latent model.
H3	H3; Table 3	Positive POQ–LF-DJA association.
H4a/H4b	H4; Table 4	Positive indirect associations in the additive latent model.
H7	H5; Table 5	Negative POQ × PsyCap coefficient for LF-DJA.
H5a/H5b	H6, POQ component; Table 5	Negative POQ × PsyCap coefficients for EE/CY in the unified equations.
H6a/H6b	H6, LF-DJA component; Table 5	EE not supported; CY nominal and near-threshold evidence (*p* = 0.0493).
H8/H8a/H8b	Complementary; Table 7	Covariate-matched restricted-model findings do not persist in the unified specification; high-minus-low intervals include zero.
H1/H2/H4/H5/H6 overall score	Auxiliary; Results and Tables 5, 7	Additive indirect association retained; POQ interaction persists, LF-DJA interaction does not; unified conditional indirect intervals include zero.
All c subhypotheses for LPE	Exploratory; Tables 3–5, 7	Distinct descriptive pattern retained; latent direct/indirect paths and unified interactions are imprecise.

Hypothesis labels were consolidated during revision. Supplementary specifications provide comparisons; Supplementary Table S21 uses the same covariates as the unified models. The revised primary conclusions follow the core SEM and unified interaction equations.

### 4.4 Sensitivity analyses

The final sample included 259 batch and 156 tracked respondents. Adding collection procedure to the observed-score additive and interaction equations changed the focal coefficients little. The joint tests of procedure interactions with the focal terms yielded *F*(3,403) = 0.01, *p* = 0.998 for LF-DJA; *F*(5,399) = 0.29, *p* = 0.919 for EE; and *F*(5,399) = 0.74, *p* = 0.590 for CY. These tests provided no clear evidence of systematic procedure-related heterogeneity, but do not demonstrate equivalence or verify the instructed time intervals in the batch subgroup.

Adjustment for the three occupational-context indicators likewise produced similar focal coefficients (Table 6). For example, the additive LF-DJA coefficients for EE and CY changed from 0.241 to 0.239 and from 0.204 to 0.205, respectively. With both procedure and occupational indicators included, the POQ × PsyCap coefficients were −0.344 for LF-DJA, −0.426 for EE, and −0.283 for CY. These checks address sensitivity to the observed sample composition; they do not establish identical paths across occupations or population representativeness.

The 21 organization clusters contained 9–34 respondents. Intercept-only random-intercept estimates yielded an ICC of 0.026 for LF-DJA and estimates near zero for EE and CY; the exploratory LPE ICC was below 0.001. CR2 standard errors with Satterthwaite degrees of freedom preserved the negative POQ × PsyCap findings for LF-DJA, EE, and CY (all *p* < 0.001). The LF-DJA × PsyCap coefficient remained nonsignificant for EE (*p* = 0.404) and close to the threshold for CY (*p* = 0.043). Small ICC estimates and these sensitivity results do not prove independence; the cluster analysis is limited by the number of organizations and by the observed-score specification. Reinstating the five endpoint-only exclusions (N = 420) left the principal observed-score interaction pattern similar: POQ × PsyCap coefficients were −0.346 for LF-DJA, −0.423 for EE, and −0.283 for CY (all *p* < 0.001). All conditional indirect and high-minus-low percentile 95% intervals continued to include zero. This check does not validate the original exclusion rule or establish the sensitivity of the latent SEM. The reinstated-case audit and full expanded-sample results are reported in Supplementary Tables S29–S31.

## 5 Discussion

This study examined employee-level associations among POQ, LF-DJA, PsyCap, and burnout outcomes in a Chinese public-sector employment context. The core latent SEM showed positive total and LF-DJA-related indirect associations of POQ with EE and CY, alongside imprecise residual direct POQ paths after simultaneous adjustment. The corresponding LPE indirect association was not supported. Complementary interaction analyses indicated consistent negative POQ × PsyCap coefficients, while LF-DJA-related moderation and conditional indirect associations were more sensitive to outcome and specification.

The findings contribute to organizational psychology in three ways. First, they connect perceived overqualification to workplace well-being by documenting a resource-underutilization appraisal pattern motivated by COR, alongside the familiar workload account of burnout. Second, consistent with SET, they distinguish capability-role appraisal from reverse-scored outcome-fairness appraisal: the two co-occur closely but remain empirically differentiable. JD-R provides an occupational-health context rather than a fully tested explanatory process. Third, they show why emotional exhaustion and cynicism should be interpreted as the primary burnout outcomes in this study, whereas overall burnout is auxiliary and LPE is exploratory.

This interpretation is deliberately narrower than a systems-level causal account. The study does not observe formal staffing rules, promotion criteria, workload allocation, managerial behavior, or actual reward distributions. Nor does it estimate feedback loops among employee appraisals and organizational arrangements. Its contribution lies in documenting employee-level diagnostic evidence: the reports of POQ, reward-recognition appraisal, PsyCap, and burnout are patterned in a theoretically coherent way. Future multilevel work can examine whether objective job-design and allocation indicators produce the same configuration and whether the configuration changes after organizational interventions.

The PsyCap findings require a differentiated interpretation. Its most consistent moderating association concerned POQ–LF-DJA. The negative POQ × PsyCap coefficients for EE and CY also persisted in unified models, but the simple slopes did not imply uniformly lower outcomes at high PsyCap. LF-DJA × PsyCap was not supported for EE and showed only near-threshold CY evidence. The conditional indirect pattern from the covariate-matched restricted specification did not persist when both outcome interaction terms were included. PsyCap is therefore associated with selected conditional relationships, without evidence here of a general protective intervention effect.

The LPE findings require particular attention. Emotional exhaustion and cynicism capture resource depletion and psychological withdrawal, whereas LPE reflects a competence-related self-evaluation. Employees who see themselves as overqualified may retain confidence in their capability even while reporting exhaustion and cynical detachment. The negative zero-order POQ–LPE association does not negate the strain findings, but its residual direct and indirect estimates in the latent model were imprecise. A competence-preserving interpretation remains an exploratory possibility rather than an established process. Future research should test whether professional identity, organization-based self-esteem, meaningful work, person-job fit, or job-crafting opportunities explain this configuration.

### 5.1 Workplace well-being implications

The practical relevance of these findings lies in workplace well-being rather than public-sector governance. They motivate consideration of credible opportunities for employees to use and develop capability, clearer recognition and developmental opportunities in relation to contribution, and feedback and resource-oriented support. The most consistent PsyCap moderation concerned the POQ–LF-DJA association, whereas its moderation of LF-DJA–burnout associations depended on the burnout outcome and model specification. Thus, the findings may inform support for employees appraising capability underuse and recognition, but do not justify treating PsyCap development as a broadly effective way to prevent burnout. PsyCap-oriented development may complement these conditions through efficacy-building feedback, goal and pathway coaching, resilience practices, and supportive supervision. Such support should not shift responsibility for capability underuse or opaque recognition onto employees.

These practices were not experimentally evaluated, so the present data do not establish that capability-use initiatives, recognition changes, or PsyCap development causally reduce burnout. PsyCap-oriented development should complement meaningful capability use, transparent recognition, and supportive work conditions, without transferring responsibility for organizational problems to employees.

### 5.2 Limitations and future research

Several limitations qualify the conclusions. Although data collection was staggered across organization-specific 14-day windows, POQ, LF-DJA, and burnout were measured together in the later survey section. The batch subgroup also lacked independently verifiable participant-level timestamps. Neither SEM nor the indirect-association estimates establish temporal or causal order. Burnout may influence work and fairness appraisals, and unmeasured work conditions may influence all three. All focal variables were self-reported, and LF-DJA was derived from distributive-justice items rather than measured as objective injustice. Its substantial overlap with POQ further limits interpretation as an independent intervening process. The cynicism factor was represented by the four-item Chinese-revised MBI-GS configuration and showed an AVE of approximately 0.473 in the revised SEM, indicating comparatively weaker convergent validity; findings involving cynicism should therefore be interpreted with corresponding measurement caution.

The non-probability convenience sample was recruited through personal contacts and was concentrated in Liaoning Province, with smaller representation from Beijing. Procedure and occupational-context sensitivity analyses yielded broadly similar observed-score estimates, but cannot establish equivalence, measurement invariance, or national representativeness. Selection processes associated with personal-contact recruitment remain unquantified. The 21-cluster CR2 analyses provide a dependence sensitivity check, while limited cluster numbers and the distinction between observed-score regressions and the latent SEM constrain their reach.

The number of hypotheses, outcomes, interactions, and specifications increases the opportunity for chance findings. No multiplicity correction was imposed, and near-threshold results should be treated cautiously. The interaction sensitivity calculation applies only to independent-observation regression under its stated assumptions; it does not establish sufficient power for all conditional indirect associations or small subgroup differences. Revised analyses were added after review. Future studies should preregister a smaller set of primary tests, retain complete screening logs and verified timestamps, and use longitudinal, multisource, and multilevel designs.

## 6 Conclusion

The evidence describes an employee-level workplace well-being pattern in which perceived capability underuse co-occurs with a less favorable distributive-justice appraisal and with emotional exhaustion and cynicism among Chinese public-sector employees. The auxiliary overall-burnout summary is consistent with the additive indirect-association pattern, whereas LPE remains exploratory. PsyCap-related moderation is outcome- and specification-dependent, and the unified model did not support the original conditional-indirect pattern. The findings support future longitudinal and multilevel research on whether changes in capability use, recognition, and personal resources are followed by changes in employee appraisals and well-being.

The contribution is deliberately bounded. The study does not establish that institutional arrangements, reward systems, or PsyCap caused the observed appraisals. It offers a transparent, theory-guided account of differentiated employee-report associations that can inform future causal and intervention research on workplace well-being.

## Data Availability

The datasets generated and analyzed for this study are not publicly available because of participant confidentiality and the restrictions of the approved ethics protocol. De-identified data may be made available by the corresponding author to qualified researchers upon reasonable request and subject to appropriate confidentiality safeguards.
