# Supplementary material for: Underused capability and burnout: distributive-justice appraisal and psychological capital among Chinese public-sector employees
Source: Front Psychol. 2026 Oct 14;17:1932134. doi: 10.3389/fpsyg.2026.1932134 (PMC13713304; doi:10.3389/fpsyg.2026.1932134)
Supplement: Supplementary file 1 [file Data_Sheet_1.pdf]

Supplementary Material

This supplement supports the revised core latent SEM and unified interaction analyses. Tables S1–S21 provide initial checks and specification comparisons. Figures S1–S6 retain the original separate-model plots. Tables S22–S28 add screening, SEM, and sensitivity details; Section 4.9 and Tables S29–S31 document the expanded-sample sensitivity analysis (N = 420). All paths denote statistical associations without establishing temporal or causal order.

1 Measurement quality, bias checks, and validity tests

Table S1. Independent-Samples t-Test Comparing Early and Late Respondents

Variable	Levene's F	Levene's p	t	df	p, two-tailed	Mean difference	Std. error difference	95% CI LL	95% CI UL
**Gender**	0.23	0.63	0.24	413.00	0.81	0.01	0.05	−0.08	0.11
**Age**	0.35	0.56	−0.43	413.00	0.67	−0.04	0.08	−0.20	0.13
**Education**	0.01	0.94	−0.30	413.00	0.77	−0.02	0.06	−0.14	0.11
**Tenure**	0.02	0.89	−0.33	413.00	0.74	−0.04	0.12	−0.26	0.19
**POQ**	0.35	0.55	0.14	413.00	0.89	0.02	0.13	−0.24	0.27
**LF-DJA**	0.19	0.66	0.68	413.00	0.50	0.10	0.14	−0.19	0.38
**EE**	1.53	0.22	−0.06	413.00	0.96	−0.01	0.14	−0.28	0.27
**CY**	0.25	0.61	−0.69	413.00	0.49	−0.09	0.13	−0.35	0.17
**LPE**	0.73	0.39	−1.04	413.00	0.30	−0.04	0.04	−0.12	0.04
**Self-efficacy**	1.22	0.27	0.03	413.00	0.97	0.00	0.13	−0.26	0.26
**Resilience**	0.94	0.33	−0.98	413.00	0.33	−0.12	0.12	−0.37	0.12
**Hope**	0.04	0.84	−0.64	413.00	0.52	−0.08	0.13	−0.33	0.17
**Optimism**	0.01	0.91	−0.45	413.00	0.65	−0.06	0.12	−0.30	0.19
**Overall burnout**	0.48	0.49	−0.55	413.00	0.58	−0.05	0.08	−0.21	0.12
**PsyCap**	0.00	1.00	−0.57	413.00	0.57	−0.06	0.11	−0.28	0.16

Note. Equal variances were assumed because all Levene's tests were nonsignificant. POQ = perceived overqualification; LF-DJA = less favorable distributive-justice appraisal based on reverse-scored distributive-justice items; EE = emotional exhaustion; CY = cynicism; LPE = low professional efficacy; PsyCap = psychological capital. Self-efficacy, resilience, hope, and optimism are the four dimensions of PsyCap. N = 415.

Table S2. Chi-Square Tests Comparing Early and Late Respondents

Variable	Pearson χ^2	df	p, two-sided
**Gender**	0.06	1	0.81
**Age**	0.29	3	0.96
**Education**	0.18	2	0.92
**Tenure**	0.15	4	1.00
**POQ**	32.81	36	0.62
**LF-DJA**	15.40	23	0.88
**EE**	38.03	30	0.15
**CY**	21.65	23	0.54
**LPE**	7.77	12	0.80
**Overall burnout**	290.20	290	0.49
**Self-efficacy**	45.15	40	0.27
**Resilience**	21.30	36	0.98
**Hope**	42.57	33	0.12
**Optimism**	34.69	32	0.34
**PsyCap**	284.33	278	0.38

Note. N = 415. Grouping uses questionnaire return order, not batch/tracked procedure. These original tests did not detect early–late differences; they do not demonstrate equivalence or eliminate nonresponse bias. Abbreviations follow the main manuscript.

Table S3. Harman's Single-Factor Test

Component	Eigenvalue	Variance explained (%)	Cumulative variance (%)
1	17.04	31.55	31.55
2	5.29	9.80	41.35
3	3.79	7.02	48.38
4	3.49	6.46	54.84
5	1.74	3.23	58.07
6	1.33	2.46	60.53

Component	Eigenvalue	Variance explained (%)	Cumulative variance (%)
7	1.08	2.00	62.53

Note. N = 415. The table reports unrotated exploratory factor-analysis results for components with eigenvalues greater than 1. The first unrotated factor explained 31.55% of total variance, below the commonly used 40% reference. This result indicates that single-factor common-method variance did not appear dominant; it does not rule out other sources of common-method or self-report bias.

Table S4. Fit Indices for Confirmatory Factor Analysis and Alternative Measurement Models

Model	Model specification	χ^2	df	χ^2/df	RMSEA	CFI	TLI	IFI	SRMR
Four-factor model	POQ, LF-DJA, overall burnout, PsyCap	2162.41	1301	1.66	0.04	0.94	0.93	0.94	0.05
Three-factor model	POQ + LF-DJA; overall burnout; PsyCap	5023.00	1374	3.66	0.08	0.74	0.73	0.74	0.09
Three-factor model	PsyCap + LF-DJA; overall burnout; POQ	5563.20	1374	4.05	0.09	0.70	0.69	0.70	0.10
Three-factor model	overall burnout + LF-DJA; PsyCap; POQ	5950.66	1374	4.33	0.09	0.67	0.66	0.67	0.14
Three-factor model	POQ + PsyCap; overall burnout; LF-DJA	6074.94	1374	4.42	0.09	0.66	0.65	0.66	0.11
Three-factor model	POQ + overall burnout; PsyCap; LF-DJA	6126.77	1374	4.46	0.09	0.66	0.64	0.66	—
Three-factor model	overall burnout + PsyCap; LF-DJA; POQ	6152.10	1374	4.48	0.09	0.66	0.64	0.66	0.11
Two-factor model	POQ + LF-DJA; overall burnout + PsyCap	6285.66	1376	4.57	0.09	0.65	0.63	0.65	0.11
Two-factor model	POQ + overall burnout; LF-DJA + PsyCap	6806.43	1376	4.95	0.10	0.61	0.59	0.61	—
Two-factor model	POQ + PsyCap; LF-DJA + overall burnout	6968.82	1376	5.07	0.10	0.60	0.58	0.60	0.15
One-factor model	POQ + LF-DJA + overall burnout + PsyCap	7745.94	1377	5.63	0.11	0.54	0.52	0.54	0.12

Note. POQ = perceived overqualification; LF-DJA = less favorable distributive-justice appraisal based on reverse-scored distributive-justice items; PsyCap = psychological capital. Overall burnout refers to the auxiliary overall-burnout summary. Lower values of χ^2/df, RMSEA, and SRMR and higher values of CFI, TLI, and IFI indicate better fit. Dashes indicate SRMR values not reported in the supplied output.

Table S5. Multicollinearity in the Unified Outcome Equations

Predictor	Tolerance	VIF
Gender	0.986	1.01
Age	0.172	5.83
Education	0.862	1.16
Tenure	0.164	6.09
POQ	0.378	2.64
LF-DJA	0.325	3.08
PsyCap	0.588	1.70
POQ × PsyCap	0.318	3.15
LF-DJA × PsyCap	0.341	2.93

Note. N = 415. Continuous focal predictors are mean-centered. All four covariates are included. Age and tenure have VIFs above 5; their individual coefficients warrant caution.

Table S6. Reliability and Initial Convergent-Validity Checks for PsyCap

PsyCap component	Cronbach's α	Initial AVE	Initial CR
Self-efficacy	0.90	0.62	0.92
Resilience	0.91	0.65	0.93
Hope	0.93	0.75	0.95
Optimism	0.90	0.67	0.93
All PsyCap items	0.96	0.52	0.96

Note. N = 415. α was recomputed from the retained item data with the four dimension labels used in the manuscript. AVE and CR retain the initial measurement-analysis values; they are not estimates from the new core SEM. New SEM loadings appear in Table S25.

Table S7. Standardized Item Loadings in the Initial Measurement Checks

Item	Initial loading	Item	Initial loading
POQ1	0.77	LPE6	0.83
POQ2	0.72	SE1	0.80
POQ3	0.73	SE2	0.81
POQ4	0.69	SE3	0.78
POQ5	0.73	SE4	0.79
POQ6	0.77	SE5	0.78
POQ7	0.78	SE6	0.77
POQ8	0.76	SE7	0.80
POQ9	0.77	RL1	0.77
LF-DJA1	0.85	RL2	0.79
LF-DJA2	0.84	RL3	0.80
LF-DJA3	0.85	RL4	0.83
LF-DJA4	0.84	RL5	0.83
EE1	0.79	RL6	0.78
EE2	0.75	RL7	0.84
EE3	0.78	HP1	0.88
EE4	0.78	HP2	0.88
EE5	0.81	HP3	0.86
CY1	0.74	HP4	0.88
CY2	0.77	HP5	0.85
CY3	0.79	HP6	0.87
CY4	0.81	OM1	0.82
LPE1	0.83	OM2	0.81
LPE2	0.78	OM3	0.80
LPE3	0.81	OM4	0.82
LPE4	0.82	OM5	0.84
LPE5	0.84	OM6	0.84

Note. Initial measurement results are retained for comparison. Table S25 gives loadings from the revised core SEM. SE = self-efficacy; RL = resilience; HP = hope; OM = optimism. Other abbreviations follow the manuscript. N = 415.

Table S8. Descriptive Statistics and Initial Validity Diagnostics

Variable	POQ	LF-DJA	EE	CY	LPE	PsyCap	Gender	Age	Education	Tenure
POQ	0.75									
LF-DJA	0.74***	0.84								
EE	0.12*	0.19***	0.78							
CY	0.12*	0.17***	0.68***	0.77						
LPE	–0.18***	–0.14**	0.01	0.02	0.82					
PsyCap	0.54***	0.54***	0.001	–0.02	–0.22***	0.72				
Gender	0.01	–0.04	0.03	0.03	–0.02	0.005	—			
Age	0.03	0.03	0.24***	0.23***	0.16**	–0.07	–0.001	—		
Education	–0.003	0.07	0.14**	0.09	0.09	0.000	0.03	0.30***	—	
Tenure	0.02	0.02	0.24***	0.22***	0.13*	–0.09	0.03	0.91***	0.35***	—
Mean	4.37	4.14	3.81	3.63	3.01	4.14	1.57	2.29	2.90	2.86
SD	1.31	1.48	1.43	1.35	0.40	1.14	0.50	0.84	0.64	1.17
Cronbach's α	0.90	0.86	0.84	0.78	0.90	0.96	—	—	—	—
Initial AVE	0.56	0.71	0.61	0.60	0.67	0.52	—	—	—	—
Initial CR	0.92	0.91	0.89	0.86	0.92	0.96	—	—	—	—

Note. N = 415. Correlations, means, SDs, and alpha coefficients are synchronized with the confirmed analytic data. Diagonals for the six focal scores are square roots of AVE from the initial measurement checks; AVE and CR retain those initial values. Main Table 2 reports the focal descriptive results, and Tables S24–S25 describe the revised latent model. All correlations use two-sided unadjusted tests: *p < .05, **p < .01, ***p < .001. Small correlations are shown to three decimals when two-decimal rounding would produce zero.

Table S9. Heterotrait–Monotrait Ratio (HTMT) Results

Variables	PsyCap	POQ	LF-DJA	EE	CY	LPE
**PsyCap**	—					
**POQ**	0.59	—				
**LF-DJA**	0.59	0.83	—			
**EE**	0.09	0.15	0.23	—		
**CY**	0.09	0.15	0.21	0.84	—	
**LPE**	0.24	0.20	0.16	0.05	0.06	—

Note. POQ = perceived overqualification; LF-DJA = less favorable distributive-justice appraisal; PsyCap = psychological capital; EE = emotional exhaustion; CY = cynicism; LPE = low professional efficacy. N = 415. All HTMT point estimates were below the conservative .85 reference. Because the POQ-LF-DJA estimate was close to this reference, its bootstrap confidence interval is reported separately in Table S10 and is interpreted together with the CFA, AVE, and construct-definition evidence.

Table S10. Heterotrait-Monotrait Ratio (HTMT) Bootstrap Confidence Intervals for Core Constructs

	Original sample (O)	Sample mean (M)	2.50%	97.50%
**PsyCap <-> Burnout**	0.22	0.24	0.18	0.30
**POQ <-> Burnout**	0.24	0.25	0.18	0.33
**POQ <-> PsyCap**	0.58	0.58	0.50	0.66
**LF-DJA <-> Burnout**	0.28	0.28	0.19	0.37
**LF-DJA <-> PsyCap**	0.59	0.59	0.50	0.67
**LF-DJA <-> POQ**	0.83	0.83	0.78	0.87

Note. POQ = perceived overqualification; LF-DJA = less favorable distributive-justice appraisal; PsyCap = psychological capital. Burnout refers to the auxiliary overall-burnout summary. The 2.50% and 97.50% columns report bootstrap percentile confidence-interval limits. The POQ-LF-DJA estimate was .83, with a 95% bootstrap confidence interval of [.78, .87].

2 Original observed-score specification comparisons

Table S11. Main and Indirect Associations on Emotional Exhaustion

Variables	M1 EE		M2 EE		M3 EE		M4 EE		M5 LF-DJA		M6 LF-DJA	
	B	SE	B	SE	B	SE	B	SE	B	SE	B	SE
**Control variables**												
**Gender**	0.09	0.14	0.09	0.14	0.11	0.14	0.12	0.14	−0.13	0.15	−0.14	0.10
**Age**	0.28	0.20	0.27	0.20	0.26	0.19	0.26	0.19	0.08	0.21	0.05	0.14
**Education**	0.15	0.11	0.15	0.11	0.12	0.11	0.11	0.11	0.16	0.12	0.18	0.08
**Tenure**	0.08	0.14	0.08	0.14	0.09	0.14	0.09	0.14	−0.05	0.15	−0.06	0.10
**Independent variable**												
**POQ**			0.13*	0.05			−0.04	0.08			0.83***	0.04
**Appraisal term**												
**LF-DJA**					0.18***	0.05	0.21**	0.07				
**R^2**	0.07		0.08		0.10		0.10		0.01		0.55	
**ΔR^2**	—		0.01		0.03		0.02		—		0.54	
**F**	7.18***		7.02***		8.96***		7.51***		0.71		99.87***	

Note. N = 415. EE = emotional exhaustion; POQ = perceived overqualification; LF-DJA = less favorable distributive-justice appraisal. The dependent variable in Models 1-4 is emotional exhaustion. The dependent variable in Models 5-6 is LF-DJA. *p < .05, **p < .01, ***p < .001. ΔR^2 indicates the increase in explained variance relative to the relevant baseline model. M2 is compared with M1; M4 is compared with M2 after adding LF-DJA to the model that already included POQ; M3 is a parallel model to M2, in which LF-DJA was entered after the control variables; M6 is compared with M5.

Table S12. Main and Indirect Associations on Cynicism

Variables	M1 CY		M2 CY		M3 CY		M4 CY		M5 LF-DJA		M6 LF-DJA	
	B	SE	B	SE	B	SE	B	SE	B	SE	B	SE
**Control variables**												
**Gender**	0.08	0.13	0.08	0.13	0.10	0.13	0.10	0.13	−0.13	0.15	−0.14	0.10
**Age**	0.35	0.19	0.35	0.19	0.34	0.18	0.34	0.18	0.08	0.21	0.05	0.14
**Education**	0.04	0.11	0.04	0.11	0.02	0.11	0.01	0.11	0.16	0.12	0.18	0.08
**Tenure**	0.01	0.14	0.01	0.14	0.02	0.14	0.02	0.14	−0.05	0.15	−0.06	0.10
**Independent variable**												
**POQ**			0.11*	0.05			−0.03	0.07			0.83***	0.04

Variables	M1 CY		M2 CY		M3 CY		M4 CY		M5 LF-DJA		M6 LF-DJA	
	B	SE	B	SE	B	SE	B	SE	B	SE	B	SE
**Appraisal term**												
**LF-DJA**					0.15***	0.04	0.17*	0.07				
**R²**	0.06		0.07		0.08		0.08		0.01		0.55	
**ΔR²**	—		0.01		0.03		0.02		—		0.54	
**F**	6.01***		5.90***		7.30***		6.09***		0.71		99.87***	

Note. N = 415. CY = cynicism; POQ = perceived overqualification; LF-DJA = less favorable distributive-justice appraisal. The dependent variable in Models 1-4 is cynicism. The dependent variable in Models 5-6 is LF-DJA. *p < .05, **p < .01, ***p < .001. ΔR^2 indicates the increase in explained variance relative to the relevant baseline model. M2 is compared with M1; M4 is compared with M2 after adding LF-DJA to the model that already included POQ; M3 is a parallel model to M2, in which LF-DJA was entered after the control variables; M6 is compared with M5.

Table S13. Main and Indirect Associations on Low Professional Efficacy

Variables	M1 LPE		M2 LPE		M3 LPE		M4 LPE		M5 LF-DJA		M6 LF-DJA	
	B	SE	B	SE	B	SE	B	SE	B	SE	B	SE
**Control variables**												
**Gender**	−0.02	0.04	−0.01	0.04	−0.02	0.04	−0.02	0.04	−0.13	0.15	−0.14	0.10
**Age**	0.13	0.06	0.13	0.06	0.13	0.06	0.13	0.06	0.08	0.21	0.05	0.14
**Education**	0.04	0.03	0.04	0.03	0.04	0.03	0.04	0.03	0.16	0.12	0.18	0.08
**Tenure**	−0.05	0.04	−0.05	0.04	−0.05	0.04	−0.05	0.04	−0.05	0.15	−0.06	0.10
**Independent variable**												
**POQ**			−0.06***	0.02			−0.04*	0.02			0.83***	0.04
**Appraisal term**												
**LF-DJA**					−0.04**	0.01	−0.01	0.02				
**R²**	0.03		0.06		0.06		0.07		0.01		0.55	
**ΔR²**	—		0.03		0.02		0.01		—		0.54	
**F**	3.30*		5.55***		4.77***		4.70***		0.71		99.87***	

Note. N = 415. LPE = low professional efficacy; POQ = perceived overqualification; LF-DJA = less favorable distributive-justice appraisal. The dependent variable in Models 1-4 is LPE. The dependent variable in Models 5-6 is LF-DJA. Higher LPE scores indicate lower professional efficacy. *p < .05, **p < .01, ***p < .001. ΔR^2 indicates the increase in explained variance relative to the relevant baseline model. M2 is compared with M1; M4 is compared with M2 after adding LF-DJA to the model that already included POQ; M3 is a parallel model to M2, in which LF-DJA was entered after the control variables; M6 is compared with M5.

Table S14. Bootstrapped Indirect Associations Specified Through Less Favorable Distributive-Justice Appraisal

Outcome variable	Association type	Estimate	SE / Boot SE	95% CI LL	95% CI UL	Result
**EE**	Total association	0.13	0.05	0.03	0.23	Significant
	Residual direct association	−0.04	0.08	−0.19	0.11	Nonsignificant
	Indirect association	0.17	0.06	0.07	0.29	Significant
**CY**	Total association	0.11	0.05	0.02	0.21	Significant
	Residual direct association	−0.03	0.07	−0.17	0.12	Nonsignificant
	Indirect association	0.14	0.05	0.03	0.25	Significant
**LPE**	Total association	−0.05	0.01	−0.08	−0.03	Significant
	Residual direct association	−0.04	0.02	−0.09	−0.001	Significant
	Indirect association	−0.01	0.02	−0.04	0.02	Nonsignificant

Note. N = 415. POQ = perceived overqualification; LF-DJA = less favorable distributive-justice appraisal; EE = emotional exhaustion; CY = cynicism; LPE = low professional efficacy. Indirect associations are specified using the POQ-LF-DJA-EE, POQ-LF-DJA-CY, and POQ-LF-DJA-LPE statistical ordering. Higher LPE scores indicate lower professional efficacy. Confidence intervals were estimated using bootstrapping. Arrow notation denotes hypothesized statistical ordering, not observed temporal sequence. Some values are reported to three decimal places because rounding to two decimal places would produce .00.

2.1 Supplementary description of dimension-level association results

At the dimension level, the association pattern differed across burnout outcomes. POQ and LF-DJA were each positively associated with emotional exhaustion, and the residual POQ association was not statistically significant after LF-DJA was entered in the joint model. Cynicism showed the same general configuration. These results are reported as indirect association patterns and do not establish that LF-DJA temporally intervenes between POQ and the outcomes.

LPE differed from emotional exhaustion and cynicism. POQ and LF-DJA were negatively associated with LPE, and the indirect association specified through LF-DJA was not statistically distinguishable from zero. Because LPE was reverse-scored, this pattern indicates that employees reporting stronger POQ may retain comparatively favorable competence self-evaluations even when they also report exhaustion and cynicism. LPE is therefore treated as an exploratory competence-related outcome rather than as a resource-depletion outcome.

Table S15. Hierarchical Regression Results for Moderation Effects on Emotional Exhaustion

Variables	M1 EE		M2 EE		M3 EE		M4 EE		M5 EE		M6 EE		M7 EE	
	B	SE	B	SE	B	SE	B	SE	B	SE	B	SE	B	SE
**Control variables**														
**Gender**	0.09	0.14	0.09	0.14	0.15	0.13	0.12	0.14	0.13	0.13	0.12	0.14	0.15	0.13
**Age**	0.28	0.20	0.28	0.20	0.31	0.18	0.27	0.19	0.28	0.19	0.27	0.19	0.31	0.18

Variables	M1 EE		M2 EE		M3 EE		M4 EE		M5 EE		M6 EE		M7 EE	
	B	SE	B	SE	B	SE	B	SE	B	SE	B	SE	B	SE
**Education**	0.15	0.11	0.16	0.11	0.12	0.11	0.12	0.11	0.11	0.11	0.12	0.11	0.12	0.11
**Tenure**	0.08	0.14	0.07	0.14	0.05	0.13	0.07	0.14	0.07	0.14	0.07	0.14	0.05	0.13
**Predictors**														
**POQ_c**			0.17**	0.06	−0.07	0.07					−0.004	0.08	−0.06	0.08
LF-DJA_c							0.24***	0.05	0.13*	0.06	0.24**	0.07	−0.01	0.08
**PsyCap_c**			−0.08	0.07	−0.03	0.07	−0.15*	0.07	−0.16*	0.07	−0.14*	0.07	−0.02	0.07
**Interaction terms**														
**POQ_c × PsyCap_c**					−0.39***	0.05							−0.43***	0.08
LF-DJA_c × PsyCap_c									−0.18***	0.05			0.04	0.06
**R^2**	0.07		0.08		0.21		0.11		0.14		0.11		0.21	
**ΔR^2**	—		0.01		0.13		0.04		0.03		0.04		0.10	
**F**	7.18***		6.07***		14.97***		8.23***		9.58***		7.03***		11.64***	

Note. N = 415. EE = emotional exhaustion; POQ = perceived overqualification; LF-DJA = less favorable distributive-justice appraisal; PsyCap = psychological capital. Variables marked with "_c" were mean-centered, and interaction terms were computed using the mean-centered variables. *p < .05, **p < .01, ***p < .001. ΔR^2 indicates the increase in explained variance relative to the relevant baseline model: M2 versus M1, M3 versus M2, M4 versus M1, M5 versus M4, M6 versus M1, and M7 versus M6. Some coefficients are reported to three decimal places to avoid rounding very small values to .00.

Table S16. Hierarchical Regression Results for Moderation Effects on Cynicism

Variables	M1 CY		M2 CY		M3 CY		M4 CY		M5 CY		M6 CY		M7 CY	
	B	SE	B	SE	B	SE	B	SE	B	SE	B	SE	B	SE
**Control variables**														
**Gender**	0.08	0.13	0.08	0.13	0.14	0.12	0.11	0.13	0.11	0.12	0.11	0.13	0.14	0.12
**Age**	0.35	0.19	0.36	0.19	0.38	0.17	0.35	0.18	0.36	0.18	0.35	0.18	0.38	0.17
**Education**	0.04	0.11	0.05	0.11	0.02	0.10	0.02	0.11	0.01	0.10	0.02	0.11	0.00	0.10
**Tenure**	0.01	0.14	−0.01	0.14	−0.03	0.13	0.00	0.13	−0.01	0.13	−0.01	0.14	−0.02	0.13
**Predictors**														
**POQ_c**	—	—	0.16**	0.06	−0.06	0.06	—	—	—	—	0.02	0.08	−0.09	0.07
**LF-DJA_c**	—	—	—	—	—	—	0.22***	0.05	0.07	0.06	0.204**	0.07	0.001	0.07

Variables	M1 CY		M2 CY		M3 CY		M4 CY		M5 CY		M6 CY		M7 CY	
	B	SE	B	SE	B	SE	B	SE	B	SE	B	SE	B	SE
PsyCap_c	—	—	−0.11	0.07	−0.06	0.06	−0.16*	0.07	−0.18**	0.06	−0.16*	0.07	−0.08	0.07
Interaction terms														
POQ_c × PsyCap_c	—	—	—	—	−0.37***	0.05	—	—	—	—	—	—	−0.28***	0.07
LF-DJA_c × PsyCap_c	—	—	—	—	—	—	—	—	−0.25***	0.04	—	—	−0.12*	0.06
R^2	0.06		0.07		0.20		0.09		0.17		0.09		0.20	
ΔR^2	—		0.02		0.12		0.04		0.07		0.03		0.11	
F	6.01***		5.36***		14.07***		7.05***		11.75***		6.04***		11.44***	

Note. N = 415. CY = cynicism; POQ = perceived overqualification; LF-DJA = less favorable distributive-justice appraisal; PsyCap = psychological capital. Variables marked with "_c" were mean-centered, and interaction terms were computed using the mean-centered variables. *$p < .05$, **$p < .01$, ***$p < .001$. ΔR^2 indicates the increase in explained variance relative to the relevant baseline model: M2 versus M1, M3 versus M2, M4 versus M1, M5 versus M4, M6 versus M1, and M7 versus M6. Some coefficients are reported to three decimal places to avoid rounding very small values to .00.

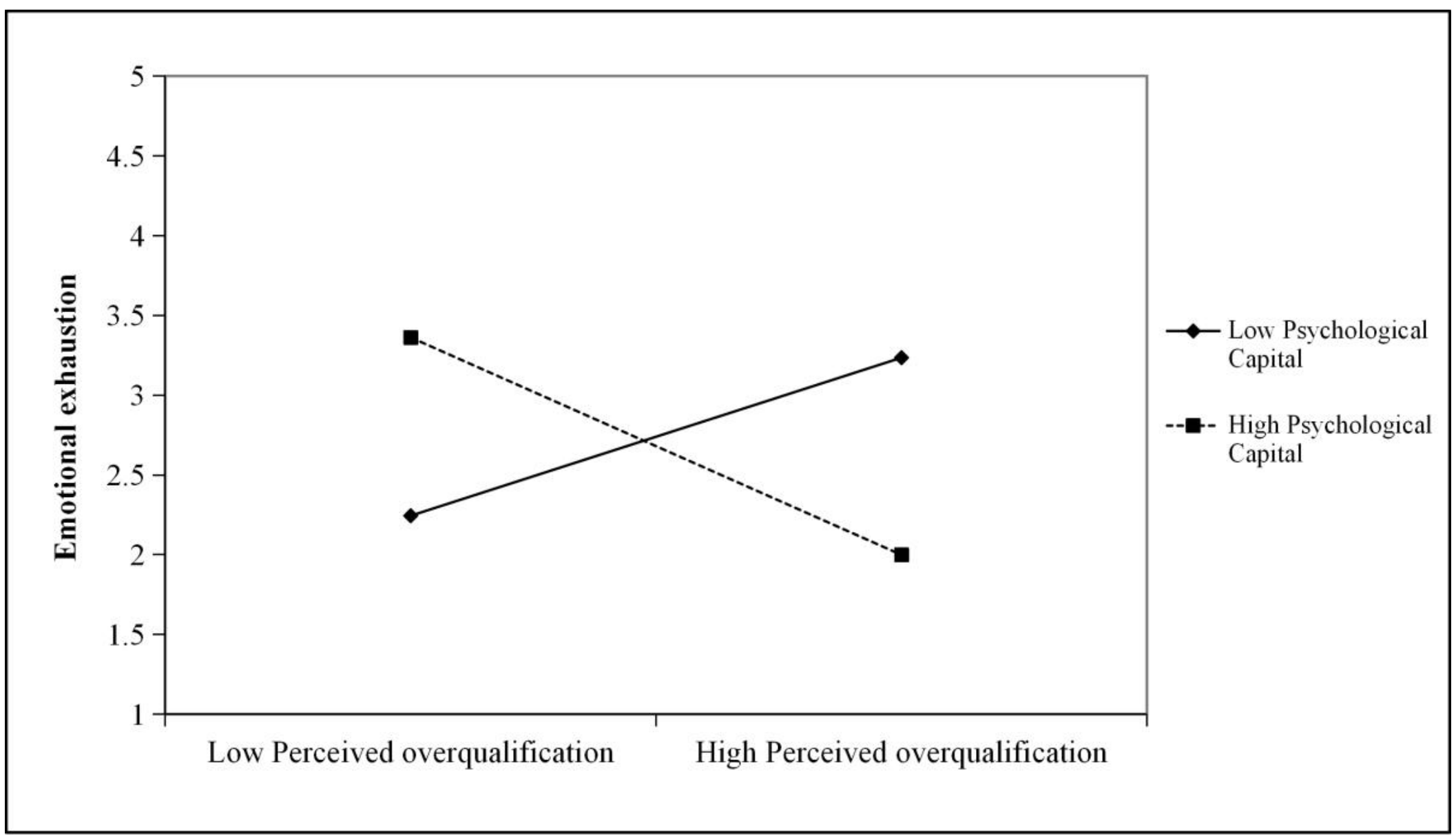

Figure S1. Conditional Association Between Perceived Overqualification and Emotional Exhaustion by Psychological Capital

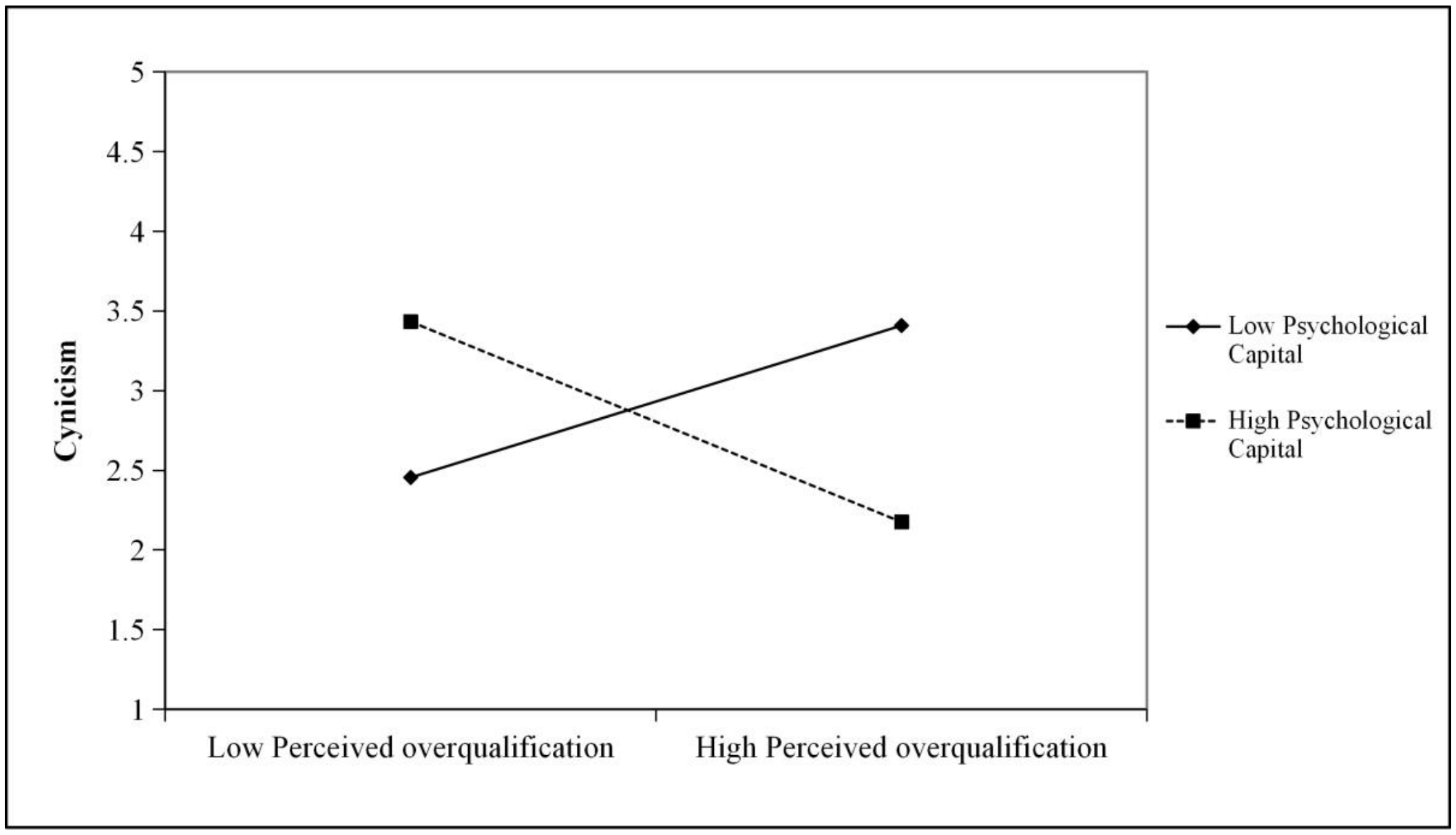

Figure S2. Conditional Association Between Perceived Overqualification and Cynicism by Psychological Capital

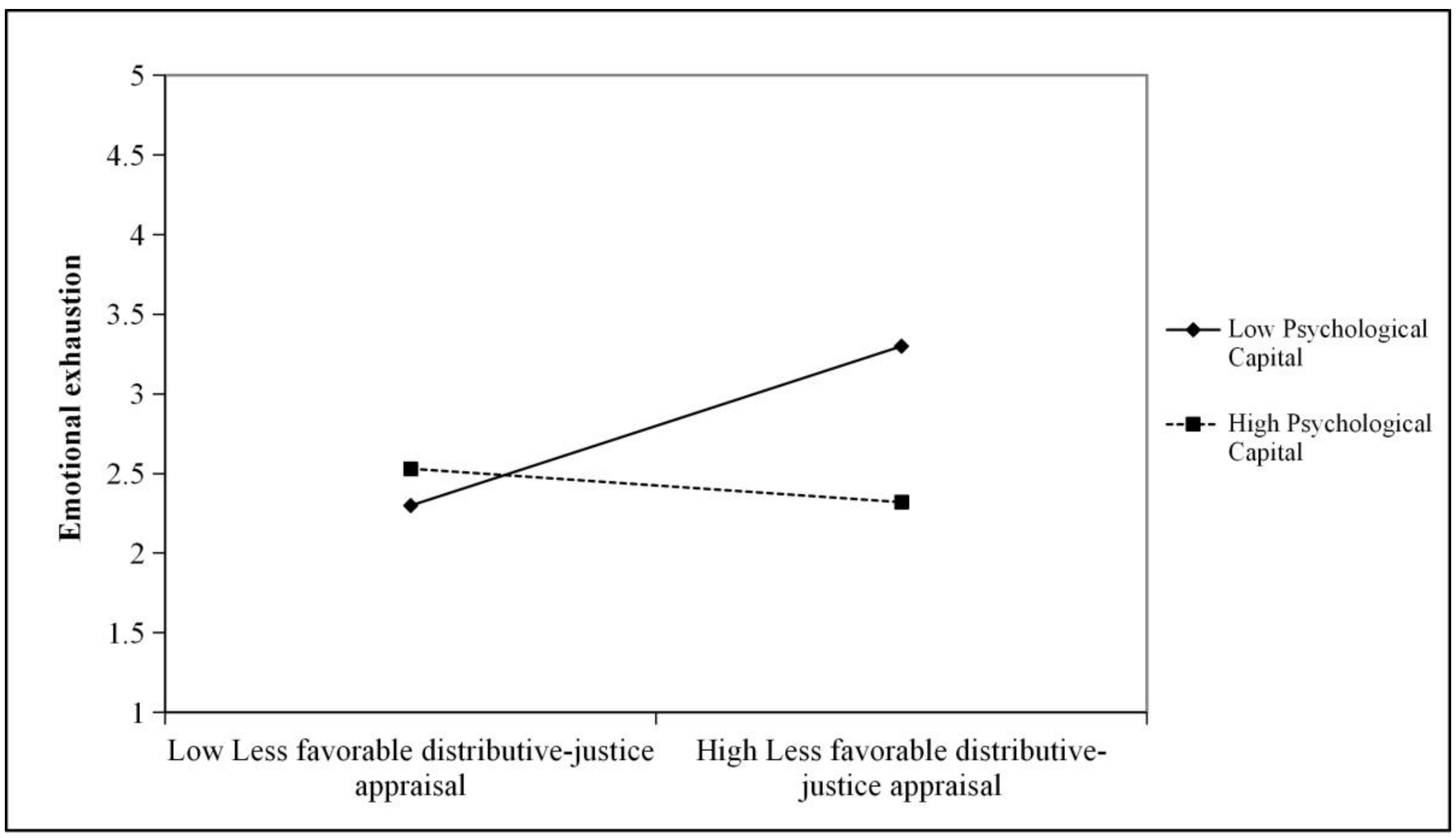

Figure S3. Conditional Association Between Less Favorable Distributive-Justice Appraisal and Emotional Exhaustion by Psychological Capital

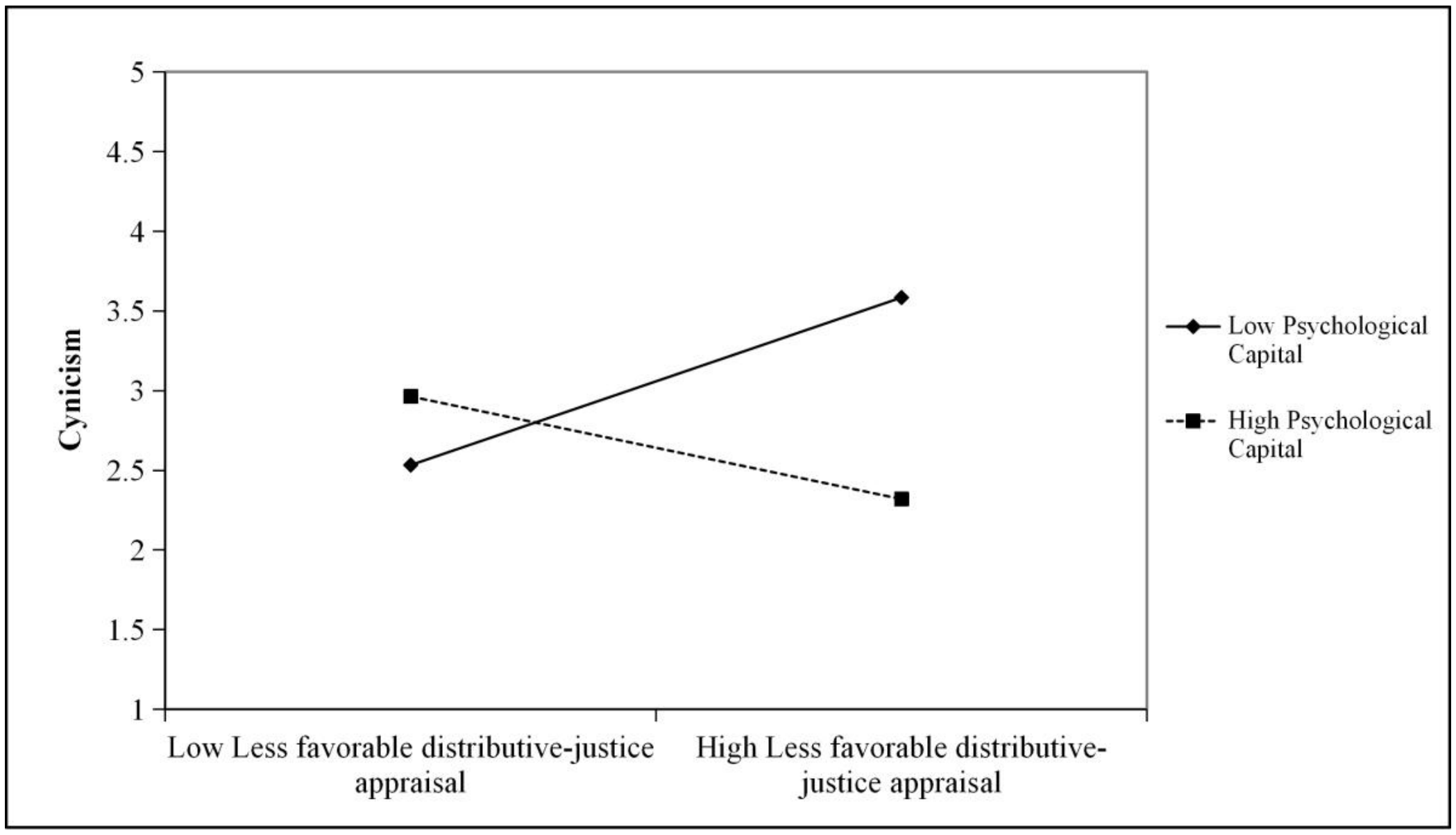

Figure S4. Conditional Association Between Less Favorable Distributive-Justice Appraisal and Cynicism by Psychological Capital

Table S17. Hierarchical Regression Results for Moderation Effects on Low Professional Efficacy

Variables	M1 LPE		M2 LPE		M3 LPE		M4 LPE		M5 LPE		M6 LPE		M7 LPE	
	B	SE	B	SE	B	SE	B	SE	B	SE	B	SE	B	SE
**Control variables**														
**Gender**	−0.02	0.04	−0.01	0.04	−0.02	0.04	−0.02	0.04	−0.02	0.04	−0.01	0.04	−0.02	0.04
**Age**	0.13	0.06	0.13	0.05	0.13	0.05	0.13	0.05	0.13	0.05	0.13	0.05	0.13	0.05
**Education**	0.04	0.03	0.04	0.03	0.04	0.03	0.04	0.03	0.05	0.03	0.04	0.03	0.04	0.03
**Tenure**	−0.05	0.04	−0.06	0.04	−0.06	0.04	−0.06	0.04	−0.06	0.04	−0.06	0.04	−0.05	0.04
**Predictors**														
**POQ_c**	—	—	−0.03	0.02	−0.01	0.02	—	—	—	—	−0.03	0.02	−0.02	0.02
**LF-DJA_c**	—	—	—	—	—	—	−0.01	0.02	0.00	0.02	0.001	0.02	0.03	0.02
**PsyCap_c**	—	—	−0.06**	0.02	−0.07***	0.02	−0.07***	0.02	−0.06***	0.02	−0.06**	0.02	−0.07**	0.02
**Interaction terms**														
**POQ_c × PsyCap_c**	—	—	—	—	0.03*	0.02	—	—	—	—	—	—	0.04	0.02
**LF-DJA_c × PsyCap_c**	—	—	—	—	—	—	—	—	0.03*	0.01	—	—	−0.001	0.02
**R^2**	0.03		0.08		0.10		0.08		0.09		0.08		0.10	
**ΔR^2**	—		0.05		0.01		0.05		0.01		0.05		0.02	

Variables	M1 LPE		M2 LPE		M3 LPE		M4 LPE		M5 LPE		M6 LPE		M7 LPE	
	B	SE	B	SE	B	SE	B	SE	B	SE	B	SE	B	SE
F	3.30*		6.21***		6.14***		5.93***		5.73***		5.31***		4.94***	

Note. N = 415. LPE = low professional efficacy; POQ = perceived overqualification; LF-DJA = less favorable distributive-justice appraisal; PsyCap = psychological capital. Higher LPE scores indicate lower professional efficacy. Variables marked with "_c" were mean-centered, and interaction terms were computed using the mean-centered variables. *p < .05, **p < .01, ***p < .001. ΔR^2 indicates the increase in explained variance relative to the relevant baseline model: M2 versus M1, M3 versus M2, M4 versus M1, M5 versus M4, M6 versus M1, and M7 versus M6. Some coefficients are reported to three decimal places to avoid rounding very small values to .00.

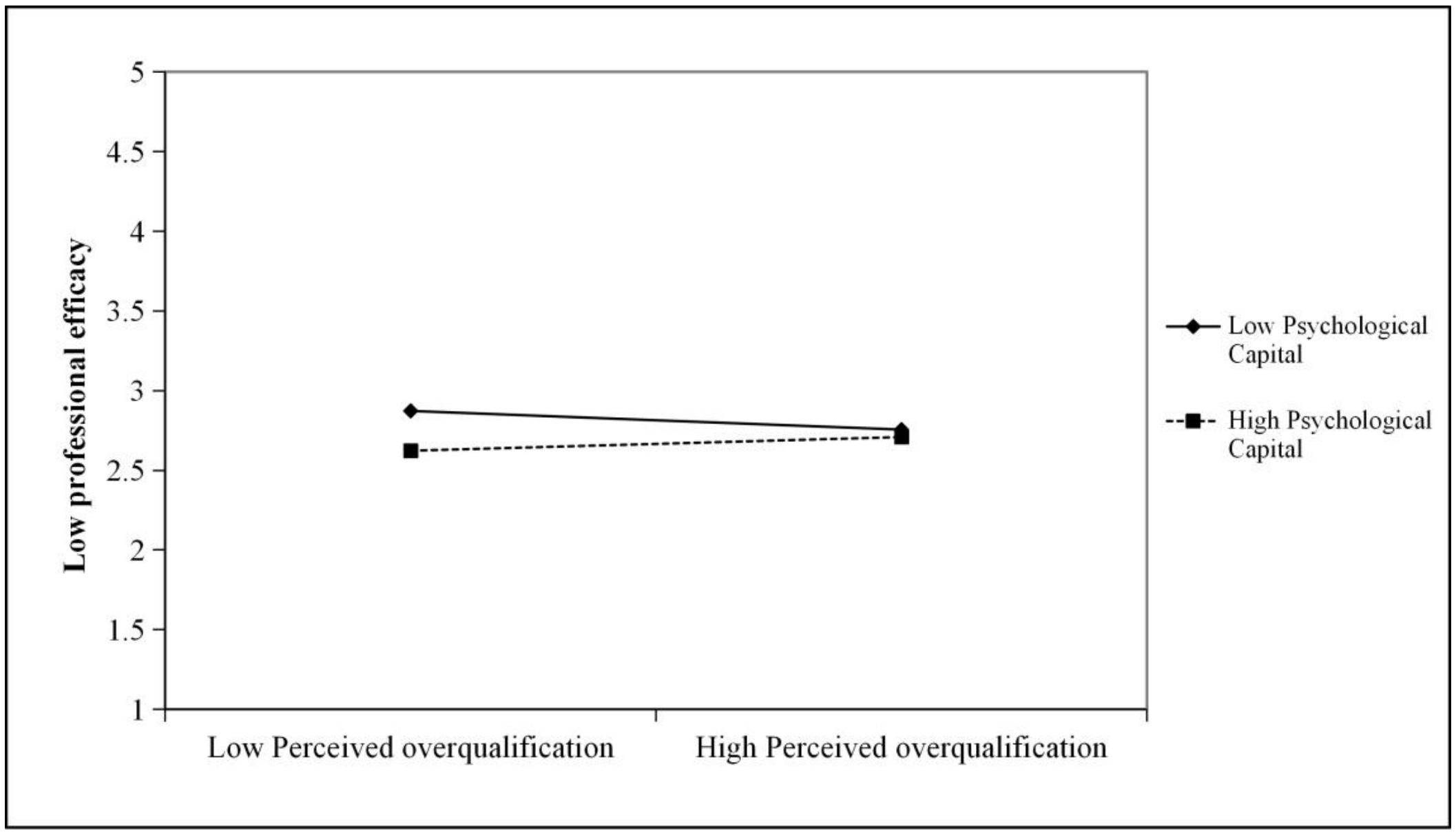

Figure S5. Conditional Association Between Perceived Overqualification and Low Professional Efficacy by Psychological Capital

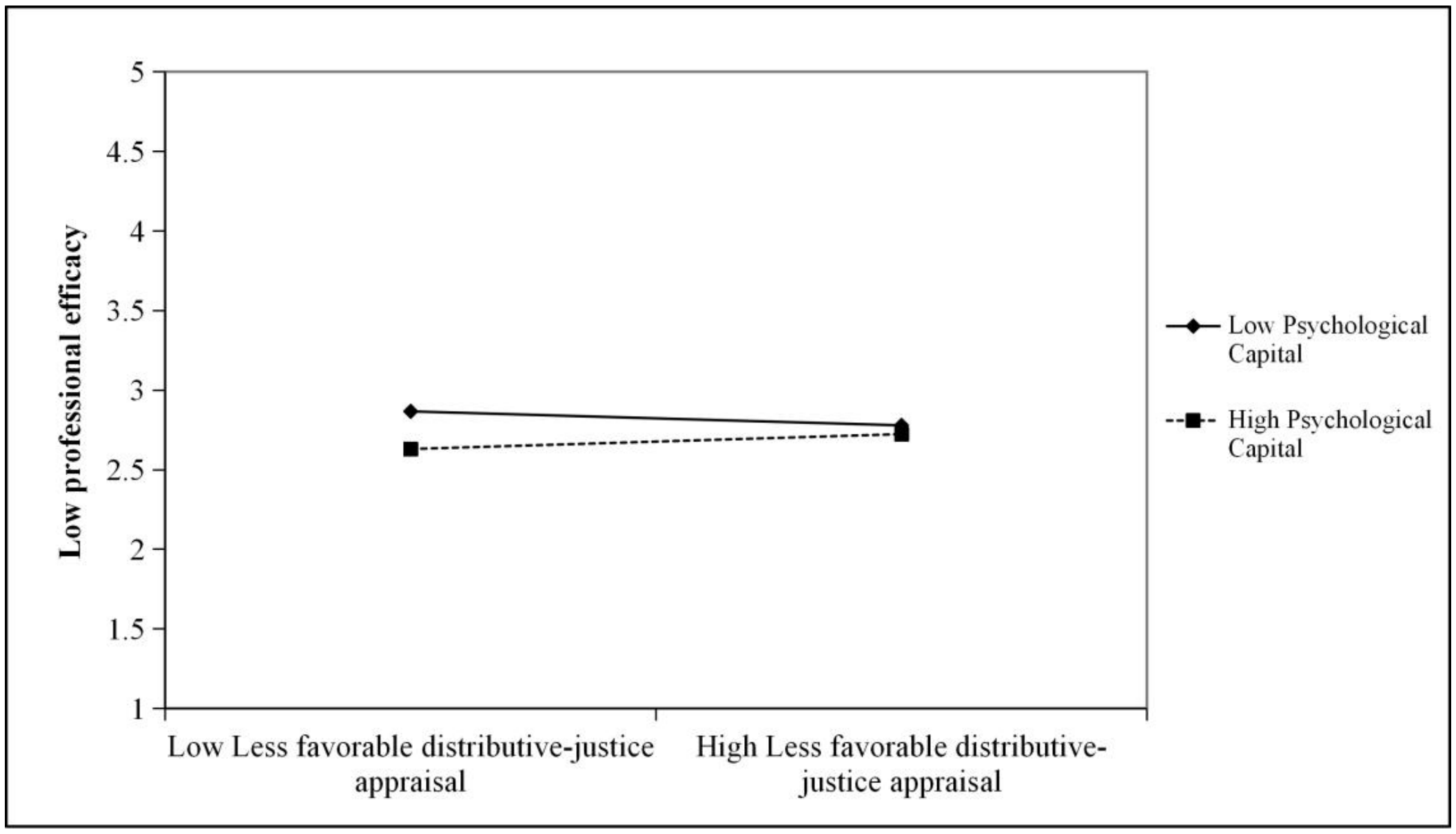

Figure S6. Conditional Association Between Less Favorable Distributive-Justice Appraisal and Low Professional Efficacy by Psychological Capital

2.2 Supplementary description of dimension-level conditional associations

The dimension-level interaction analyses indicated that PsyCap conditioned several associations involving POQ, LF-DJA, emotional exhaustion, and cynicism. In separate models, the positive POQ and LF-DJA associations with emotional exhaustion and cynicism were weaker at higher PsyCap. This pattern is consistent with a conditional employee-resource interpretation, while not demonstrating that PsyCap causally changes work conditions or prevents burnout.

For LPE, the pattern differed. In separate models, interaction terms were positive and statistically significant. Because LPE was reverse-scored, these interactions indicated that the negative POQ-LPE and LF-DJA-LPE associations were weaker at higher PsyCap. However, when both interaction terms were included in the full model, neither remained statistically significant, whereas the main PsyCap association remained significant and negative. PsyCap therefore showed a more stable direct association with professional efficacy than a robust conditional association in the joint model.

Table S18. PROCESS Model 1 Results: Conditional Associations Involving Psychological Capital

Conditioned association	Interaction term	B	SE	95% CI LL	95% CI UL	ΔR^2	F	Result
POQ → EE	POQ × PsyCap	−0.39	0.05	−0.49	−0.30	0.12	62.82	Significant
POQ → CY	POQ × PsyCap	−0.37	0.05	−0.46	−0.28	0.12	61.57	Significant
POQ → LPE	POQ × PsyCap	0.03	0.01	0.01	0.06	0.01	5.32	Significant
LF-DJA → EE	LF-DJA × PsyCap	−0.18	0.05	−0.27	−0.09	0.03	15.91	Significant
LF-DJA → CY	LF-DJA × PsyCap	−0.25	0.04	−0.33	−0.17	0.07	36.31	Significant
LF-DJA → LPE	LF-DJA × PsyCap	0.03	0.01	0.001	0.05	0.01	4.20	Significant

Note. N = 415. POQ = perceived overqualification; LF-DJA = less favorable distributive-justice appraisal; PsyCap = psychological capital; EE = emotional exhaustion; CY = cynicism; LPE = low professional efficacy. Higher LPE scores indicate lower professional efficacy. The reported coefficients are unstandardized interaction estimates from PROCESS Model 1. CI = confidence interval; LL = lower limit; UL = upper limit. Estimates are described as statistically distinguishable from zero when the 95% CI does not include zero.

Table S19. PROCESS Model 7 Results: First-Stage Conditional Indirect Associations Involving Psychological Capital

**Outcome variable**	Conditional indirect-association specification	**Moderator**	**Index**	**SE**	**95% CI LL**	**95% CI UL**	**Result**
EE	POQ-LF-DJA- EE	POQ × PsyCap	−0.07	0.03	−0.12	−0.02	Significant
CY	POQ-LF-DJA- CY	POQ × PsyCap	−0.06	0.02	−0.10	−0.01	Significant
LPE	POQ-LF-DJA- LPE	POQ × PsyCap	0.005	0.01	−0.01	0.02	Nonsignificant

Note. N = 415. POQ = perceived overqualification; LF-DJA = less favorable distributive-justice appraisal; PsyCap = psychological capital; EE = emotional exhaustion; CY = cynicism; LPE = low professional efficacy. Higher LPE scores indicate lower professional efficacy. The reported values are bootstrapped indices of conditional indirect association from PROCESS Model 7. In this specification, PsyCap conditions the POQ-LF-DJA association. CI = confidence interval; LL = lower limit; UL = upper limit. Estimates are described as statistically distinguishable from zero when the 95% CI does not include zero. Arrow notation does not establish temporal sequence.

Table S20. Model 14 Indices of Moderated Mediation

Outcome	Index	Boot SE	95% bootstrap CI	Interval excludes zero
EE	–0.164	0.037	[–0.238, –0.093]	Yes
CY	–0.227	0.033	[–0.292, –0.162]	Yes
LPE	0.020	0.011	[–0.002, 0.043]	No

Note. N = 415. The index is a × b3: a is the POQ coefficient in the LF-DJA equation; b3 is the LF-DJA × PsyCap coefficient in the outcome equation. Both equations control gender, age, education, and tenure. Boot SE and percentile 95% CIs use 5,000 case resamples (seed 20260918). These are unstandardized moderated-mediation indices, not interaction coefficients. The statistical label does not establish causal mediation or temporal order.

Table S21. Restricted Model 58 with the Same Covariates as the Unified Models

Outcome	Quantity	PsyCap level	Estimate	SE / Boot SE	95% CI
EE	POQ × PsyCap → LF-DJA	—	–0.342	0.033	[–0.407, –0.276]
EE	LF-DJA × PsyCap → EE	—	–0.199	0.047	[–0.292, –0.106]
EE	Conditional indirect association	Low (–1 SD)	0.364	0.073	[0.226, 0.514]
EE	Conditional indirect association	Mean (0)	0.091	0.035	[0.025, 0.162]
EE	Conditional indirect association	High (+1 SD)	–0.005	0.013	[–0.039, 0.016]
CY	POQ × PsyCap → LF-DJA	—	–0.342	0.033	[–0.407, –0.276]
CY	LF-DJA × PsyCap → CY	—	–0.274	0.044	[–0.360, –0.188]
CY	Conditional indirect association	Low (–1 SD)	0.389	0.065	[0.266, 0.520]
CY	Conditional indirect association	Mean (0)	0.062	0.035	[–0.004, 0.133]
CY	Conditional indirect association	High (+1 SD)	–0.021	0.019	[–0.065, 0.008]
LPE	POQ × PsyCap → LF-DJA	—	–0.342	0.033	[–0.407, –0.276]
LPE	LF-DJA × PsyCap → LPE	—	0.024	0.014	[–0.003, 0.051]
LPE	Conditional indirect association	Low (–1 SD)	–0.017	0.022	[–0.062, 0.026]
LPE	Conditional indirect association	Mean (0)	0.004	0.011	[–0.017, 0.025]
LPE	Conditional indirect association	High (+1 SD)	0.004	0.004	[–0.003, 0.015]

Note to Table S21. N = 415. Both LF-DJA and outcome equations control gender, age, education, and tenure using the same coding as the unified models. Focal scores and mean centering are identical. The LF-DJA equation contains POQ, PsyCap, and POQ × PsyCap; the outcome equation contains POQ, LF-DJA, PsyCap, and LF-DJA × PsyCap. Relative to the unified models, only POQ × PsyCap is omitted from the outcome equation. The common first-stage interaction is repeated for each outcome for readability. Product-term SEs and 95% CIs are ordinary OLS estimates with t-based intervals. Conditional indirect estimates are (a1 + a3w)(b1 + b3w), where w is centered PsyCap; their Boot SEs and percentile CIs use the same 5,000 case resamples as main Table 7 (seed 20260918). Low and high levels use the unrounded sample SD (1.1396259693). All values denote associations without established temporal order.

2.3 Specification dependence of conditional indirect associations

In the covariate-matched restricted Model 58, the conditional indirect association through LF-DJA was distinguishable from zero for EE at low and mean PsyCap and for CY at low PsyCap. The same sample, covariates, scores, centering, moderator levels, and bootstrap resamples were used as in the unified models. This comparison isolates the inclusion of POQ × PsyCap in the outcome equation; it does not establish a temporal or causal sequence.

In the unified equations containing both outcome interactions, every conditional indirect interval and high-minus-low interval included zero for EE, CY, LPE, and auxiliary overall burnout (main Table 7; Supplementary Table S28). Thus, the conditional-indirect pattern in the covariate-matched restricted comparison did not persist in the unified specification. Positive indirect associations in the additive core SEM concern a different specification.

3 Interpretive boundaries and construct distinctions

POQ is an employee appraisal that one's qualifications exceed role requirements; LF-DJA is a reverse-scored appraisal that received outcomes are less commensurate with contribution. POQ concerns capability–role fit, whereas LF-DJA concerns outcome fairness. The initial CFA and discriminant-validity checks (Tables S4 and S8–S10) support retaining differentiated but highly related measures. The POQ–LF-DJA HTMT estimate was .83 with a bootstrap 95% interval [.78, .87]. The upper bound slightly exceeds .85 but remains below 1.00. Accordingly, the two appraisals are modeled separately and interpreted cautiously. Neither construct verifies objective job requirements, pay allocation, promotion decisions, or organizational fairness.

The non-probability Chinese public-sector sample provides a study context rather than population representativeness. COR motivates the resource-underutilization account and social exchange theory specifies the contribution–outcome appraisal. JD-R provides broader occupational-health context; conventional job demands and job resources were not directly tested. PsyCap does not replace meaningful capability use, transparent recognition, or supportive work conditions.

4 Supplementary methodological details

4.1 Sampling and collection procedures

Guided by the employee-level framework, the study examined reports of POQ, LF-DJA, PsyCap, and burnout outcomes. To reduce item-context effects and respondent burden, the study used a two-section completion design with partial and instructed temporal separation (Weng & McElroy, 2012). Data collection proceeded on a rolling basis from March 17 to April 20, 2025, with each participating organization using its own 14-day field window. The first section included demographic characteristics, control variables, and PsyCap; the later section included POQ, distributive-justice items, and burnout. Participants were instructed to complete the sections in sequence, typically 2–12 days apart. This separation distinguished PsyCap from the later-section variables but did not separate POQ, LF-DJA, and burnout from one another.

Participants were recruited from 21 organizations: seven universities, five state-owned enterprises, four hospitals, and five government agencies. Organizations were approached through the authors' existing personal contacts and referrals, without formal organization-level eligibility criteria. The employee-level eligibility condition was current formal employment in a participating organization; no additional age, education, tenure, or rank restrictions were imposed. Employees were invited primarily through existing personal contacts and participated voluntarily. Managers were not informed of individual participation decisions and did not have access to individual responses.

The organizations were located primarily in Dandong and Shenyang, Liaoning Province, with smaller representation from Benxi and Beijing. The seven universities covered these four cities; the state-owned enterprises, hospitals, and government agencies were located in Dandong and Shenyang. This was a non-probability, multi-organization convenience field sample with limited national geographic coverage.

In the batch procedure, 336 questionnaire sets were distributed and 288 were returned. Both sections were distributed together with instructions on order and timing; participant-level timestamps were unavailable. In the tracked procedure, 179 respondents completed the first section in person and 174 matched responses were obtained at the second section through non-identifying codes. Tracked respondents received a small low-value token of appreciation. These procedures yielded 462 returned questionnaires; the final analytic sample included 259 batch and 156 tracked respondents. Return-order labels used in Tables S1–S2 are distinct from collection-procedure labels.

4.2 Data screening and mutually exclusive exclusion categories

Questionnaires were excluded when they met the study's data-quality screening criteria. Substantial missingness was defined as missing responses on at least 19 of the 54 focal measurement items (≥35% of the measurement battery). Excessive endpoint responding was defined as responding with either endpoint of the 7-point scale (1 or 7) on at least 39 of the 54 measurement items (≥72.2%), after excluding cases already identified as straight-line or deterministic patterned responding. Exclusion categories were applied as mutually exclusive first-exclusion classifications to avoid double counting. In the screening workbook, the straight-line category comprised records with the same stored value on all 54 focal items. Supplementary Tables S22–S23 report the screening criteria and case-level audit. All 415 retained cases had complete focal-item data.

During revision, the patterned-response classifications were audited in stored workbook column order: POQ (9 items), LF-DJA (4), EE (5), CY (4), professional efficacy (6), and the four PsyCap dimensions (7, 7, 6, and 6). This is the audit

order, not the order of questionnaire administration. Four templates exactly matched the first 22 and final 26 stored items, restarting at the beginning of each segment: an ascending 1–7 cycle (four records), a descending 7–1 cycle (four), an alternating 1–7 sequence (three), and a repeating 2–2–6–6 sequence (three). The corresponding six-item professional-efficacy sequences were exactly 3–4–2–3–4–2, 3–2–4–3–2–4, 2–4–2–4–2–4, and 4–4–2–2–4–4, respectively. The last sequence is a six-item prefix of a period-four sequence, not two complete repetitions. These patterns describe the stored screening records; they should not be equated with sequences of raw response options without reference to the original scoring records.

The 462-return worksheet reconciles exactly with the 47 excluded records and the confirmed 415-case analytic dataset on all 54 focal items, four demographic variables, and the return-order label. Patterned cases were classified before applying the endpoint criterion; three alternating-pattern cases also met the endpoint threshold and were counted only in the patterned category. No additional exclusion category was created. The five endpoint cases had 39, 40, 41, 42, or 44 endpoint responses; these counts were recalculated directly from the item cells.

Table S22. Operational Screening Criteria and Exclusion Counts

First-exclusion category	Operational criterion	n
Straight-line responding	Same stored value on all 54 focal items in the screening workbook	16
Deterministic patterned responding	One of four specified templates exactly matches the first 22 and final 26 items in stored column order; the intervening six-item sequence matches its stated template	14
Substantial missingness	At least 19 of 54 focal items missing (≥35%)	12
Excessive endpoint responding	At least 39 of 54 responses equal to 1 or 7 (≥72.2%), after straight-line/patterned classification	5
Total excluded	Each questionnaire counted once	47
Retained analytic sample	No missing focal-item values; no case meets the endpoint threshold	415

Pattern templates occurred in 4, 4, 3, and 3 cases, respectively. The 12 missingness cases had 19–31 missing items; the screening threshold was ≥19.

Table S23. Case-Level Screening Audit

Audit ID	Worksheet row	Recorded category	Missing items	Endpoint items
E01	2	Patterned	0	14
E02	3	Straight-line	0	0
E03	4	Straight-line	0	0
E04	5	Substantial missingness	26	3
E05	6	Substantial missingness	31	2
E06	7	Patterned	0	14
E07	8	Substantial missingness	26	2
E08	9	Straight-line	0	0
E09	10	Patterned	0	48
E10	11	Substantial missingness	20	4
E11	12	Patterned	0	0
E12	13	Excessive endpoints	0	44
E13	14	Straight-line	0	0
E14	15	Excessive endpoints	0	42
E15	16	Straight-line	0	0
E16	17	Patterned	0	14
E17	18	Substantial missingness	27	6
E18	19	Patterned	0	14
E19	20	Patterned	0	48
E20	21	Patterned	0	0
E21	22	Patterned	0	14
E22	23	Straight-line	0	0
E23	24	Straight-line	0	0
E24	25	Straight-line	0	0
E25	26	Patterned	0	14
E26	27	Excessive endpoints	0	40
E27	28	Patterned	0	48
E28	29	Patterned	0	0
E29	30	Substantial missingness	25	6
E30	31	Straight-line	0	0
E31	32	Straight-line	0	0
E32	33	Substantial missingness	29	3
E33	34	Substantial missingness	19	6
E34	35	Substantial missingness	28	2
E35	36	Straight-line	0	0
E36	37	Straight-line	0	0
E37	38	Straight-line	0	0

Audit ID	Worksheet row	Recorded category	Missing items	Endpoint items
E38	39	Excessive endpoints	0	39
E39	40	Substantial missingness	20	4
E40	41	Excessive endpoints	0	41
E41	42	Straight-line	0	0
E42	43	Patterned	0	14
E43	44	Straight-line	0	0
E44	45	Straight-line	0	0
E45	46	Substantial missingness	31	3
E46	47	Substantial missingness	29	4
E47	48	Patterned	0	14

Audit IDs are display labels for this table, not original participant identifiers. Worksheet row refers to the supplied exclusion sheet, whose header is row 1. Missing and endpoint counts use only the 54 focal item cells. Endpoint counts are shown before applying category precedence; cases already classified as patterned are not counted again. No item responses or organizational identifiers are disclosed.

4.3 Source versions and Chinese-language adaptation

For POQ and distributive justice, the original English instruments were used as source versions during questionnaire development, and study-specific Chinese versions were prepared using a forward-back translation procedure. This choice reflected the questionnaire-development procedure and should not be interpreted as implying that previously published Chinese adaptations were invalid.

For burnout, the administered 15-item configuration was based on the Chinese MBI-GS revision reported by Li and Shi (2003), comprising five EE items, four CY items, and six professional-efficacy items. Li and Shi revised the original 16-item MBI-GS (Schaufeli et al., 1996); in their revision, one cynicism item (Item 13 in the initial 16-item Chinese questionnaire) was removed because of substantial cross-loading in exploratory factor analysis. The resulting 5-4-6 structure therefore predated the present study and was adopted during questionnaire development, before data collection or analysis. The original English MBI-GS was retained as a reference for item content and construct meaning when the administered Chinese wording was checked. No burnout item was removed or selected on the basis of the current sample's reliability, factor loadings, AVE, or SEM results.

POQ used the nine-item Maynard et al. (2006) instrument; distributive justice used four Colquitt (2001) items, scored so that higher LF-DJA represents a less favorable appraisal. PsyCap used Zhang et al.'s (2010) Chinese 26-item Positive Psychological Capital Questionnaire: self-efficacy (7), resilience (7), hope (6), and optimism (6). In observed-score analyses, PsyCap is the equally weighted mean of the four dimension means; auxiliary overall burnout is the equally weighted mean of EE, CY, and LPE. The confirmed analysis workbook already contains analysis-direction scores. The CY factor showed an AVE of approximately .473 in the revised SEM; this comparatively weaker convergent validity is acknowledged as a measurement limitation.

4.4 Core latent SEM specification and inference

The primary analysis was an item-level latent-variable structural equation model of the core direct and indirect associations. POQ, LF-DJA, emotional exhaustion, cynicism, and LPE were specified as distinct first-order factors. The four PsyCap dimensions were modeled as first-order factors loading on a second-order PsyCap factor. All 54 measurement items were retained. EE and CY were primary outcomes; LPE was exploratory. Overall burnout was analyzed separately as an auxiliary composite and was not entered alongside its components as another latent outcome.

The structural equations regressed LF-DJA on POQ, PsyCap, and the four covariates, and each burnout dimension on POQ, LF-DJA, PsyCap, and the same covariates. Exogenous variables covaried freely, and the three outcome disturbances were allowed to covary. Item-error covariances and residual covariances among the first-order PsyCap dimensions were fixed to zero. Marker indicators fixed the first loading of each first-order factor to one; the self-efficacy loading on second-order PsyCap was also fixed to one. Covariates retained their original numerical coding. The covariance model contained 58 observed variables and 161 free parameters.

Normal-theory maximum likelihood was used for covariance estimation, treating item responses as continuous. Direct-path inference used observed-information sandwich standard errors and two-sided Wald tests. Indirect and total associations used percentile 95% intervals from 5,000 case-resampled bootstrap fits, with the same model refitted in each sample. All 5,000 fits converged. Conventional, unscaled ML fit indices are reported. The core structural component is saturated conditional on the measurement structure and covariates; fit therefore assesses the measurement restrictions and does not identify the direction of the POQ–LF-DJA–burnout ordering. Additional measurement specifications and loadings are provided in Supplementary Tables S24–S25.

In structural notation, LF-DJA is regressed on POQ, PsyCap, gender, age, education, and tenure. EE, CY, and LPE are each regressed on POQ, LF-DJA, PsyCap, and those same four covariates. The residual covariances among EE, CY, and LPE are freely estimated. Each item loads only on its designated factor. The first-order PsyCap factors load only on the second-order PsyCap factor. There are no cross-loadings or correlated item errors in this revised core model. The covariance parameterization and the recursive structural equations yield the same fitted covariance structure.

Table S24. Revised Core SEM Specification and Fit

Quantity	Value
Analytic sample	415
Observed variables	54 focal items + 4 observed covariates = 58
Free covariance-model parameters	161
Degrees of freedom	1,550
χ^2; χ^2/df	2,512.253; 1.621
CFI; TLI	.935; .931
RMSEA and 90% CI	.0387 [.0359, .0414]
SRMR	.0459
Smallest item-error variance	.2717 in the internally standardized item metric
Successful case bootstrap fits	5,000 of 5,000; seed 20260919
Direct-path inference	Observed-information sandwich SEs and normal Wald tests
Indirect/total inference	5,000-fit percentile bootstrap intervals

These are conventional, unscaled maximum-likelihood fit indices. Sandwich inference is not an MLR-scaled χ^2 correction. The core structural component is saturated conditional on the specified measurement model and covariates; fit cannot identify the direction of the associations. Main Tables 3–4 and Figure 2 report the direct, indirect, and total paths.

Table S25. Standardized Loadings in the Revised Core SEM

Item	Standardized loading	Item	Standardized loading
POQ1	0.724	LPE6	0.809
POQ2	0.693	SE1	0.788
POQ3	0.687	SE2	0.771
POQ4	0.637	SE3	0.728
POQ5	0.682	SE4	0.745
POQ6	0.737	SE5	0.749
POQ7	0.748	SE6	0.699
POQ8	0.731	SE7	0.762
POQ9	0.729	RL1	0.732
LF-DJA1	0.764	RL2	0.755
LF-DJA2	0.766	RL3	0.748
LF-DJA3	0.779	RL4	0.804
LF-DJA4	0.817	RL5	0.788
EE1	0.728	RL6	0.726
EE2	0.672	RL7	0.813
EE3	0.731	HP1	0.853
EE4	0.699	HP2	0.846
EE5	0.760	HP3	0.832
CY1	0.630	HP4	0.849
CY2	0.680	HP5	0.816
CY3	0.701	HP6	0.835
CY4	0.736	OM1	0.776
LPE1	0.793	OM2	0.772
LPE2	0.721	OM3	0.756
LPE3	0.746	OM4	0.784
LPE4	0.762	OM5	0.793
LPE5	0.818	OM6	0.805

SE = self-efficacy; RL = resilience; HP = hope; OM = optimism. Other abbreviations follow the manuscript. These loadings come from the same model as main Figure 2 and differ from the initial measurement checks in Table S7.

Second-order PsyCap loading	Standardized estimate
Self-efficacy	0.869
Resilience	0.908
Hope	0.844
Optimism	0.878

4.5 Unified interactions and specification comparisons

Complementary observed-score interaction models used mean-centered POQ, LF-DJA, and PsyCap. The LF-DJA equation included POQ, PsyCap, POQ × PsyCap, and the four covariates. Each outcome equation included POQ, LF-DJA, PsyCap, POQ × PsyCap, LF-DJA × PsyCap, and the same covariates. This unified specification estimates both outcome interactions together, rather than combining coefficients from separate models. Conditional indirect associations were computed as the product of the conditional POQ–LF-DJA and LF-DJA–outcome coefficients at mean PsyCap and at one SD below and above the mean; high-minus-low differences were bootstrapped with 5,000 shared case resamples.

PROCESS Models 1, 4, 7, 14, and 58 provide complementary specification comparisons in the Supplementary Material. Table S20 reports Model 14 moderated-mediation indices, defined as the POQ–LF-DJA coefficient multiplied by the LF-DJA × PsyCap coefficient, with bootstrap SEs and intervals. This statistical index does not establish causal mediation. The restricted Model 58 comparison in Table S21 was re-estimated using the same 415 cases, gender, age, education, tenure, score construction, centering, PsyCap levels, and 5,000 case resamples as the unified models. Both equations include the four covariates. The LF-DJA equation is identical to the unified specification; only POQ × PsyCap is omitted from the outcome equation. Original analyses used SPSS 24.0, AMOS 24.0, SmartPLS 4.0, and PROCESS 4.2. The additional SEM, bootstrap, regression, and sensitivity computations were implemented in Python 3.12 with NumPy 2.3 and SciPy 1.17. These revision-stage analyses were not preregistered.

In the unified LF-DJA equation, the conditional POQ coefficient is the POQ coefficient plus the POQ × PsyCap coefficient multiplied by centered PsyCap. In each unified outcome equation, the conditional LF-DJA coefficient is the LF-DJA coefficient plus the LF-DJA × PsyCap coefficient multiplied by centered PsyCap. Their product defines the conditional indirect association. Both outcome products are included in the same fitted outcome equation. For each of 5,000 shared case resamples (seed 20260918), both equations are refitted and the product and high-minus-low contrast recomputed.

4.6 Collection procedure, occupational context, and organization clustering

Procedure sensitivity analyses added a batch/tracked indicator and tested procedure interactions with the focal predictors. Occupational-context sensitivity analyses added indicators for state-owned enterprise, healthcare, and government employment, using university employment as the reference. Organization membership was assessed using random-intercept ICC estimates and bias-reduced cluster-robust regression standard errors (CR2) with coefficient-specific Satterthwaite degrees of freedom. Given 21 organizations, this small-sample adjustment was preferred to unadjusted large-sample cluster inference (Pustejovsky & Tipton, 2018). These observed-score analyses assess sensitivity to sample context and dependence; they do not constitute multigroup latent measurement-invariance tests. Detailed procedure, occupational-context, and cluster results are provided in Supplementary Tables S26–S27.

The group-size summaries and procedure heterogeneity tests appear in Table S26. Adding context indicators changes the focal coefficients little (main Table 6). Table S27 gives CR2 inference for the interaction terms, using the actual 21 organizations as clusters. The adjustment uses an identity working covariance and the within-cluster residual adjustment based on the inverse square root of the corresponding residual-maker block. Coefficient-specific Satterthwaite degrees of freedom account for the small number of clusters (Pustejovsky & Tipton, 2018). Case-bootstrap latent SEM intervals remain based on individual resampling; the observed-score cluster analyses are complementary sensitivity checks, not cluster-robust latent SEM inference.

Table S26. Collection Procedure and Sample Context

Collection procedure	n	Occupational context	n
Batch	259	University	182
Tracked	156	State-owned enterprise	98
		Healthcare	74
		Government	61

Organization	n	Organization	n	Organization	n
G01	16	H03	17	U01	34
G02	14	H04	14	U02	31
G03	12	S01	25	U03	28
G04	10	S02	22	U04	26
G05	9	S03	20	U05	24
H01	23	S04	17	U06	21
H02	20	S05	14	U07	18

Organization codes are non-identifying analytic labels. U, S, H, and G denote university, state-owned enterprise, hospital, and government settings, respectively. Organization sizes range from 9 to 34.

Outcome equation	Procedure omnibus F	df1	df2	p	Random-intercept ICC
LF-DJA	0.013	3	403	.998	0.026
EE	0.289	5	399	.919	<.001
CY	0.745	5	399	.590	<.001
LPE	1.740	5	399	.124	<.001
Overall burnout (auxiliary)	0.828	5	399	.530	<.001

The omnibus test adds procedure-by-focal-term products to equations that already include procedure. LF-DJA has three such products; each outcome equation has five. These nonsignificant tests do not establish equivalence, verified batch timing, or measurement invariance. ICCs are intercept-only REML estimates.

Table S27. Organization-Cluster Sensitivity of Unified Interaction Terms

Outcome	Product term	B	CR2 SE	df	CR2 p	95% CR2 CI
LF-DJA	POQ × PsyCap	−0.342	0.026	16.09	<.001	[−0.397, −0.287]
EE	POQ × PsyCap	−0.426	0.059	13.74	<.001	[−0.554, −0.299]
EE	LF-DJA × PsyCap	0.040	0.047	13.63	.404	[−0.060, 0.141]
CY	POQ × PsyCap	−0.280	0.067	13.74	<.001	[−0.424, −0.136]
CY	LF-DJA × PsyCap	−0.117	0.053	13.63	.043	[−0.230, −0.004]
LPE	POQ × PsyCap	0.044	0.022	13.74	.061	[−0.002, 0.091]
LPE	LF-DJA × PsyCap	−0.001	0.021	13.63	.969	[−0.045, 0.043]
Overall burnout	POQ × PsyCap	−0.221	0.039	13.74	<.001	[−0.305, −0.136]
Overall burnout	LF-DJA × PsyCap	−0.026	0.026	13.63	.332	[−0.081, 0.029]

N = 415 in 21 organization clusters. CR2 applies to the baseline unified observed-score models. Degrees of freedom are coefficient-specific Satterthwaite approximations. Multiplicity is not adjusted; the CY LF-DJA × PsyCap finding remains near the conventional threshold. CR2 intervals are distinct from the ordinary OLS intervals in main Table 5.

Table S28. Conditional Indirect Associations in the Unified Specification

Outcome	PsyCap level or contrast	Estimate	Boot SE	95% bootstrap CI
EE	Low (–1 SD)	–0.045	0.083	[–0.204, 0.119]
EE	Mean	–0.002	0.035	[–0.071, 0.067]
EE	High (+1 SD)	0.005	0.013	[–0.019, 0.035]
EE	High – low	0.050	0.083	[–0.115, 0.209]
CY	Low (–1 SD)	0.120	0.084	[–0.039, 0.293]
CY	Mean	0.001	0.036	[–0.069, 0.073]
CY	High (+1 SD)	–0.015	0.016	[–0.050, 0.011]
CY	High – low	–0.134	0.084	[–0.305, 0.026]
LPE	Low (–1 SD)	0.025	0.034	[–0.047, 0.089]
LPE	Mean	0.014	0.012	[–0.011, 0.037]
LPE	High (+1 SD)	0.003	0.004	[–0.004, 0.013]
LPE	High – low	–0.022	0.034	[–0.085, 0.048]
Overall burnout	Low (–1 SD)	0.033	0.049	[–0.060, 0.131]
Overall burnout	Mean	0.004	0.021	[–0.037, 0.047]
Overall burnout	High (+1 SD)	–0.002	0.008	[–0.020, 0.014]
Overall burnout	High – low	–0.036	0.048	[–0.132, 0.058]

N = 415. Both outcome interactions are included. Table S21 uses the same four covariates, scores, centering, PsyCap levels, and bootstrap resamples, but omits POQ × PsyCap from the outcome equation. Percentile intervals use 5,000 shared case resamples. Low and high PsyCap are ±1 sample SD (1.1396) from its mean. Every level-specific and high-minus-low interval includes zero. Values are identical to main Table 7; rounding does not change inference.

4.7 Power, precision, multiplicity, and bias checks

An a priori power calculation was not documented. A revision-stage noncentral-F sensitivity calculation for one incremental interaction term, N = 415, $\alpha = .05$, and nine predictors in the full outcome equation indicated minimum detectable f^2 values of .0190 at 80% power and .0254 at 90% power. These calculations assume independent observations and concern a single regression interaction, not the power of the full SEM or conditional indirect association. Smaller interactions may remain undetected, and inference emphasizes interval width rather than observed post-hoc power.

The sensitivity calculation uses a noncentral F distribution with one numerator degree of freedom, 405 residual degrees of freedom, and noncentrality parameter $N \times f^2$. The full observed-score outcome equation has nine predictors plus an intercept. This calculation does not account for clustering, test an observed post-hoc power estimate, or establish adequate power for the full latent model, all conditional indirect associations, or subgroup comparisons.

All significance tests were two-sided with $\alpha = .05$. No formal multiplicity correction was imposed. Interpretation considered effect magnitude, confidence intervals, the distinction among primary, auxiliary, and exploratory outcomes, and consistency across specifications. Borderline p values were not treated as strong evidence, and $p < .10$ was not used as a separate significance category. A nonsignificant coefficient was interpreted as insufficient evidence, not as evidence of a zero association or equivalence.

Early–late comparisons (Tables S1–S2) use return order and cannot demonstrate the absence of nonresponse bias. Harman’s first unrotated component and the one-factor comparison (Tables S3–S4) suggest that one common factor was not dominant, but cannot exclude other common-method or self-report bias. The VIF analysis in Table S5 includes

the entire unified outcome design; age and tenure require particular caution. The POQ–LF-DJA HTMT point estimate of .83 and interval [.78, .87] support retaining related but differentiated appraisals with substantial overlap; these diagnostics do not establish an independent causal process.

4.8 Ethics and data availability

The study involving human participants was reviewed and approved by the Ethics Committee of Northeastern University, China (Protocol Code: NEU-BA-202503-006; Approval Date: 5 March 2025). All participants received written study information and provided informed consent before completing the survey. Participation was voluntary, and participants could discontinue without penalty.

The datasets generated and analyzed for this study are not publicly available because of participant confidentiality and the restrictions of the approved ethics protocol. De-identified data may be made available by the corresponding author to qualified researchers upon reasonable request and subject to appropriate confidentiality safeguards.

4.9 Expanded-sample sensitivity after reinstating endpoint-only exclusions

This revision-stage sensitivity analysis reinstated the five complete questionnaires excluded only for excessive endpoint responding. These records are identified in Table S29 using the same display audit IDs and source worksheet rows as Table S23. No straight-line, patterned-response, or substantial-missingness exclusions were reinstated. The expanded sample comprised 420 cases; the primary analytic sample and latent SEM remain N = 415.

The stored analysis-direction item scores were used without another reversal. POQ and LF-DJA are means of nine and four items, respectively; EE, CY, and LPE are means of five, four, and six items. PsyCap is the equally weighted mean of the four dimension means (7, 7, 6, and 6 items), and auxiliary overall burnout is the equally weighted mean of EE, CY, and LPE. The same four covariates—gender, age, education, and tenure—retained their original numerical coding. POQ, LF-DJA, and PsyCap were centered within the expanded sample. Both outcome interaction terms were included simultaneously.

The LF-DJA equation included POQ, PsyCap, POQ × PsyCap, and the four covariates. Each burnout equation included POQ, LF-DJA, PsyCap, POQ × PsyCap, LF-DJA × PsyCap, and the four covariates. Ordinary least-squares estimation used conventional model-based standard errors, two-sided t tests, and 95% t intervals, with 412 residual degrees of freedom for LF-DJA and 410 for each burnout outcome. Table S30 reports every focal coefficient and the corresponding N = 415 coefficient for comparison; covariates and intercepts were included in estimation but are not displayed.

Table S29. Reinstated Records in the Expanded-Sample Sensitivity Analysis

Audit ID	Worksheet row	Missing items	Endpoint items	Reason for original exclusion
E12	13	0	44	Endpoint-only exclusion
E14	15	0	42	Endpoint-only exclusion
E26	27	0	40	Endpoint-only exclusion
E38	39	0	39	Endpoint-only exclusion
E40	41	0	41	Endpoint-only exclusion

Source: the supplied 47-case exclusion worksheet (header in row 1). Counts were recomputed from the 54 focal item cells. All five records are complete and originally met the threshold of at least 39 endpoint responses. Audit IDs are display labels, not participant identifiers.

Table S30. Focal Unified-Model Coefficients After Reinstating Five Records

Outcome	Predictor	B (415)	B (420)	SE (420)	95% CI (420)	p (420)
LF-DJA	POQ	0.500	0.491	0.043	[0.407, 0.575]	<.001
LF-DJA	PsyCap	0.305	0.313	0.045	[0.225, 0.401]	<.001
LF-DJA	POQ × PsyCap	−0.342	−0.346	0.033	[−0.411, −0.281]	<.001
EE	POQ	−0.058	−0.090	0.077	[−0.243, 0.062]	.245
EE	LF-DJA	−0.005	0.011	0.075	[−0.137, 0.159]	.888
EE	PsyCap	−0.018	−0.008	0.073	[−0.151, 0.135]	.914
EE	POQ × PsyCap	−0.426	−0.423	0.077	[−0.574, −0.272]	<.001
EE	LF-DJA × PsyCap	0.040	0.030	0.063	[−0.093, 0.153]	.633
CY	POQ	−0.090	−0.105	0.073	[−0.249, 0.038]	.151
CY	LF-DJA	0.001	0.011	0.071	[−0.129, 0.150]	.881
CY	PsyCap	−0.075	−0.069	0.069	[−0.204, 0.066]	.316
CY	POQ × PsyCap	−0.280	−0.283	0.073	[−0.425, −0.140]	<.001
CY	LF-DJA × PsyCap	−0.117	−0.118	0.059	[−0.234, −0.002]	.0455
LPE	POQ	−0.020	−0.018	0.023	[−0.063, 0.027]	.438
LPE	LF-DJA	0.027	0.027	0.022	[−0.017, 0.071]	.220
LPE	PsyCap	−0.073	−0.073	0.022	[−0.115, −0.030]	<.001
LPE	POQ × PsyCap	0.044	0.041	0.023	[−0.004, 0.086]	.072
LPE	LF-DJA × PsyCap	−0.001	0.003	0.019	[−0.034, 0.039]	.880
Overall burnout	POQ	−0.056	−0.071	0.046	[−0.161, 0.019]	.122
Overall burnout	LF-DJA	0.008	0.016	0.045	[−0.071, 0.104]	.716
Overall burnout	PsyCap	−0.055	−0.050	0.043	[−0.135, 0.035]	.248
Overall burnout	POQ × PsyCap	−0.221	−0.221	0.046	[−0.311, −0.132]	<.001
Overall burnout	LF-DJA × PsyCap	−0.026	−0.029	0.037	[−0.101, 0.044]	.441

N = 420. B is unstandardized. All equations include gender, age, education, and tenure. Each outcome equation includes both product terms. B (415) reproduces the baseline coefficients in main Table 5. P values and intervals use conventional OLS t inference without multiplicity adjustment; they are not CR2 or latent-SEM estimates.

The POQ × PsyCap coefficients remained negative for LF-DJA (B = −.346), EE (B = −.423), and CY (B = −.283), all p < .001. LF-DJA × PsyCap remained nonsignificant for EE and close to the conventional threshold for CY. The findings continue to warrant caution about multiplicity, weak coefficients, and model dependence.

Conditional indirect associations were computed as (a1 + a3W) × (b1 + b3W), using the conditional POQ coefficient from the LF-DJA equation and the conditional LF-DJA coefficient from the outcome equation. The expanded-sample PsyCap mean was 4.1414 and its sample SD was 1.1357. Low, mean, and high PsyCap therefore correspond to centered values of –1 SD, 0, and +1 SD. The high-minus-low contrast is the difference between the high- and low-PsyCap products.

For each of 5,000 shared case resamples (seed 20260922), both equations were refitted to the same resampled cases and all products and contrasts recomputed. Centering constants and evaluation levels were fixed at the expanded-sample values. Bootstrap standard errors are the sample standard deviations of the bootstrap estimates; intervals are the 2.5th and 97.5th percentiles. Table S31 reports all four outcomes and all levels and contrasts.

Table S31. Expanded-Sample Conditional Indirect Associations

Outcome	PsyCap level / contrast	Estimate	Boot SE	95% bootstrap CI
EE	Low (–1 SD)	–0.021	0.089	[–0.190, 0.164]
EE	Mean	0.005	0.035	[–0.061, 0.074]
EE	High (+1 SD)	0.004	0.012	[–0.018, 0.033]
EE	High – low	0.025	0.089	[–0.160, 0.196]
CY	Low (–1 SD)	0.128	0.083	[–0.028, 0.295]
CY	Mean	0.005	0.035	[–0.063, 0.074]
CY	High (+1 SD)	–0.012	0.015	[–0.047, 0.012]
CY	High – low	–0.140	0.084	[–0.308, 0.020]
LPE	Low (–1 SD)	0.021	0.034	[–0.050, 0.086]
LPE	Mean	0.013	0.012	[–0.010, 0.037]
LPE	High (+1 SD)	0.003	0.004	[–0.004, 0.012]
LPE	High – low	–0.018	0.034	[–0.083, 0.053]
Overall burnout	Low (–1 SD)	0.043	0.049	[–0.051, 0.143]
Overall burnout	Mean	0.008	0.021	[–0.034, 0.049]
Overall burnout	High (+1 SD)	–0.002	0.007	[–0.019, 0.013]
Overall burnout	High – low	–0.045	0.049	[–0.145, 0.050]

N = 420; 5,000 case resamples; percentile 95% intervals. All level-specific and high-minus-low intervals include zero. These estimates address sensitivity to the five endpoint-only exclusions in the unified observed-score equations. They do not validate the exclusion rule or establish the sensitivity of the latent SEM.

5 Interpretation of low professional efficacy

LPE is an exploratory competence-related outcome. Its negative zero-order associations with POQ and LF-DJA differ from the positive EE/CY associations, but its residual direct and indirect estimates in the core latent model are imprecise. In the unified interaction equation, neither product term meets the .05 threshold. PsyCap has a negative main association with LPE, without a supported conditional-indirect pattern. A competence-preserving interpretation is therefore a possibility for future research rather than an established explanation or evidence against the primary exhaustion and cynicism results.

6 References

Colquitt, J. A. (2001). On the dimensionality of organizational justice: a construct validation of a measure. Journal of Applied Psychology, 86(3), 386-400. https://doi.org/10.1037/0021-9010.86.3.386

Li, C. P., & Shi, K. (2003). The influence of distributive justice and procedural justice on job burnout. Acta Psychologica Sinica, 35(5), 677-684.

Maynard, D. C., Joseph, T. A., & Maynard, A. M. (2006). Underemployment, job attitudes, and turnover intentions. Journal of Organizational Behavior, 27(4), 509-536. https://doi.org/10.1002/job.389

Pustejovsky, J. E., & Tipton, E. (2018). Small-sample methods for cluster-robust variance estimation and hypothesis testing in fixed effects models. Journal of Business & Economic Statistics, 36(4), 672-683. https://doi.org/10.1080/07350015.2016.1247004

Schaufeli, W. B., Leiter, M. P., Maslach, C., & Jackson, S. E. (1996). Maslach Burnout Inventory - General Survey. In C. Maslach, S. E. Jackson, & M. P. Leiter (Eds.), The Maslach Burnout Inventory (3rd ed.) - Test Manual. (pp. 19-26). Consulting Psychologists Press.

Weng, Q., & McElroy, J. C. (2012). Organizational career growth, affective occupational commitment and turnover intentions. Journal of Vocational Behavior, 80(2), 256-265. https://doi.org/10.1016/j.jvb.2012.01.014

Zhang, K., Zhang, S., & Dong, Y. (2010). Positive Psychological Capital: Measurement and Relationship with Mental Health. Studies of Psychology and Behavior, 8(01), 58-64. https://doi.org/10.3969/j.issn.1672-0628.2010.01.011
